# Energy consumption and intestinal microbiome disorders of yellow catfish *(Pelteobagrus fulvidraco)* under cold stress

**DOI:** 10.3389/fphys.2022.985046

**Published:** 2022-09-13

**Authors:** Junru Hu, Hongxia Zhao, Guoxia Wang, Yuping Sun, Lei Wang

**Affiliations:** ^1^ Guangdong Key Laboratory of Animal Breeding and Nutrition, Key Laboratory of Animal Nutrition and Feed Science in South China of Ministry of Agriculture and Rural Affairs, Institute of Animal Science, Guangdong Academy of Agricultural Sciences, Guangzhou, China; ^2^ Key Laboratory of Ecology and Environment Science in Guangdong Higher Education, Guangdong Provincial Key Laboratory for Healthy and Safe Aquaculture, College of Life Science, South China Normal University, Guangzhou, China; ^3^ Institute of Modern Aquaculture Science and Engineering, South China Normal University, Guangzhou, China

**Keywords:** *Pelteobagrus fulvidraco*, cold stress, energy, transcriptome, metabolome analyses, intestinal microbiome

## Abstract

The yellow catfish (*P. fulvidraco*), as one of the economically-relevant freshwater fish found in China, cannot tolerate cold stress. Understanding the physiological and biochemical mechanisms under cold stress may provide insights for improving yellow catfish management in the cold. Therefore, we investigated the metabolic and intestinal microbiota changes in cold stress in response to induced cold stress. We found that cold stress in yellow catfish lead to a significant increase in the consumption of glucose and triglycerides, as well as increased use of cholesterol as an alternate energy source. Moreover, cold stress also activated several significant biological processes in the fish such as thermogenesis, oxidative phosphorylation, the spliceosome machinery, RNA transport, protein processing that occurs in the ER, and purine and pyrimidine metabolism pathways involved in energy production. On the other hand, many other mechanisms like insulin resistance, starch and sucrose metabolism, and the glyoxylate and dicarboxylate metabolic pathways that also served as energy production pathways were weakened. Furthermore, organic acids and their derivatives as well as the lipids and lipid-like molecules were mainly altered in cold stress; prenol lipids, steroids, and their derivatives were significantly upregulated, while fatty acyls and glycerophospholipids were significantly downregulated. Transcriptomic and metabolomic integrated analysis data revealed that carbohydrate metabolism, lipid metabolism, amino acid metabolism, and nucleotide metabolism were involved in cold stress resistance. In addition, the intestinal microbiota abundance was also reduce and the pathogenic bacteria of plesiomonas was rapidly appreciation, which suggesting that cold stress also impaired intestinal health. This research study could offer insights into winter management or the development of feed to promote cold resistance in yellow catfish.

## Introduction

The temperature of the water is considered a significant environmental factor that affects the survival of warm-water fish. Acute or chronic changes in water temperature caused negative effects on various physiological processes, including altered metabolism, reduced food intake, attenuated mobility, and stagnant growth, and are a substantial cause of their mortality. Therefore, understanding the effects of cold stress has consistently been one of the main focuses of the aquaculture industry. Aquatic species have evolved sophisticated, effective, and versatile adaptive strategies to tolerate the cold temperatures, including variations in metabolic rate, enzyme activity, body composition, and energy generation (Cheng et al., 2013; Dietrich et al., 2018; Tan et al., 2020), as a multi-level and multi-functional system (Kang, 2020).

Currently, RNA-Seq (Transcriptomic profiling) is an effective approach for investigating the genome and functional gene responses of aquatic animals to various abiotic and biotic stresses (Zhang et al., 2018; Sun et al., 2019). Metabolomics focuses primarily on the global identification and quantification of different endogenous metabolic products that are generated by the cells, tissues, organs, or organisms at any given time period (Weckwerth, 2003; Jin et al., 2020). Thus, an integrated approach using RNA-Seq and metabolomics can provide a better understanding of biological responses in cells and organs (Saito and Matsuda, 2010; Patti et al., 2012). Furthermore, intestinal microbiome analyses have provided fundamental information on how the environment modulates fish intestinal microbiota and subsequently, health (Huyben et al., 2018; Cruz-Flores et al., 2022a). 16S rRNA metagenomics has been used to identify the genomes of several microorganisms without culturing but by analyzing the hypervariable regions of the 16S rRNA gene, which has commonly been applied to study fish intestinal microbiomes (Handelsman, 2004).

Yellow catfish (*P. fulvidraco*) is a warm-temperature fish that is widely farmed in the central and the southern areas of China. With the success of artificial breeding techniques, the production of yellow catfish in 2020 reached 56.55 × 10^7^ tons , accounting for a huge market in Japan, South Korea, Southeast Asia, and other countries. The yellow catfish usually grows in freshwater between 20°C and 32°C. The optimum growth temperature is 26.8°C and temperatures below 19°C lead to reduce food intake and growth performance in yellow catfish (Qiang, 2019). In our previous studies, we found that serum protein, glucose, and triglyceride levels were altered in response to cold stress (Hu et al., 2019); however, the physiological mechanisms underlying this change were not known. Here, the researchers used various techniques like RNA-Seq, metabolomics, and 16S rRNA analyses for identifying the signaling pathways, metabolomic changes, and intestinal microbiome associated with cold stress responses in yellow catfish, which may be valuable in determining opportunities by using feeds specially formulated for the colder seasons, to improve the nutritional status and metabolism rate of the fish.

## Materials and methods

### Experimental animals and samples

Healthy yellow catfish were obtained from Guangdong Liang’s Aquatic Seed Industry Co. LTD (Foshan, China). Fish were acclimated to the experimental conditions for 1 week at a water temperature of 27 ± 0.5°C, a pH of 7.0–7.5, and a dissolved oxygen concentration >6 mg L^−1^, NH4^+^ of <0.2 mg L^−1^. The catfish were provided a commercial diet twice a day, each 6% per kg body weight. After acclimation, fish were subjected to cold stress in a temperature-controlled aquaculture system (DaLian Huixin Titanium Equipment Development Co., Ltd) at the Guangdong Academy of Agricultural Sciences. Then, the researchers selected 120 healthy fish (initial weight 12.5 ± 0.05 g) and divided them randomly into 2 groups: the treatment (T0 group) and control (G0 group) groups. Three tanks (each containing 170 L of water) as one repetition of a group were used for this purpose. The fish in the G0 group was maintained at a temperature of 27 ± 0.5°C and the T0 group was maintained at temperatures progressively decreasing by 1°C/1 h from 27 ± 0.5°C to 13 ± 1°C. The fish were fasted for the duration of the experiment but did not die.

At a temperature of 13 ± 1°C, the fish were alive but could not swim. Blood, liver, and hind intestine were sampled from both groups. The researchers collected the blood samples from the caudal veins of the 12 fish in each tank. The collected blood was divided into two sets, wherein the blood samples that were collected from six fish each were placed in one blood collection tube and considered as one sample. The samples were centrifuged at 4°C, at a rate of 4,000 rpm for 10 min, and stored at –80°C until further biochemical and metabolomic analyses. The liver and hind intestine of three fish per tank were extracted, snap-frozen using liquid nitrogen, and stored at –80°C until further analyses.

### Biochemical analysis

Serum biochemical indices were evaluated using a fully automated biochemical analyzer (HITACHI 7170A), with the help of commercial kits (NJBI Clinical Reagent, Nanjing, China) following the kit instructions.

### RNA-seq analysis and data annotation

The researchers extracted the Total RNA from the liver of the fish using TRIzol (Invitrogen), after which they extracted the total genomic DNA sample from the fish using the DNase I (TaKaRa). They further examined the integrity and the concentration of the RNA samples using the Agilent 2100 Bioanalyzer (Agilent Technologies, Santa Clara, CA, United States) and the NanoDrop™ 8000 Spectrophotometer (Thermo Scientific, MS, United States), respectively. The RNA samples with OD260/280 nm = 1.8–2.2 and integrity ≥7 (total >2 μg) were considered for further analysis (Lu et al., 2015). The researchers established an RNA library using the TruSeqTM RNA Sample Preparation Kit (Illumina, SD, CA, United States). Firstly, they enriched the poly-A-tailed mRNA from the total RNA sample (1 μg) using magnetic beads that contained the Oligo dT. Then, this mRNA was randomly divided into smaller fragments (≈200 bps) using a fragmentation Buffer. They used the SuperScript double-stranded cDNA Synthesis Kit (Invitrogen, MS, United States) for adding 6-base random primers (Illumina, SD, CA, United States) for synthesizing the first cDNA strand using the reverse transcription technique, where mRNA was used as a template. Thereafter, the second strand was synthesized. Both the strands formed a stable double-stranded structure. The double-stranded cDNAs possess a sticky end, hence, the researchers added the end-repair mix for achieving a flat end. Then, they added the A base in excess to the 3′ end for connecting the Y-shaped connector, as per kit instructions. After cDNA enrichment by PCR, the DNA Clean Beads were used for screening the bands with a 200–300 bp size, with the help of TBS380 (Picogreen). The researchers sequenced the complete library that was developed using the Noveseq 6000 sequencing platform (Illumina, SD, CA, United States) for high-throughput sequencing. They finally captured the paired-end reads of length PE150. Sequencing was commissioned to Majorbio Biomedical Technology Co., Ltd (Shanghai, China).

The image files obtained by the Noveseq 6000 sequencing platform were converted into raw data using Illumina’s CASAVA base identification and analysis software. The raw data were subjected to decoupling and low-quality data filtering and screening, and high-quality clean data was obtained. We then assembled the clean data using the Cufflinks software and mapped them to the reference genome. In this study, the researchers used the Transcripts Per Million reads (TPM) for determining the gene expression. They used the DEGseq2 software package for determining the presence of differentially expressed genes (DEGs). The screening threshold was set at a fold change >2 and *P*-adjust was set as <0.05, wherein the smaller the value of the P-adjust, the more significant the difference in the gene expression. They further used the Goatools software for conducting the Gene Ontology (GO) functional annotation and the Kyoto Encyclopedia of Genes and Genomes (KEGG) pathway enrichment analyses of the DEGs, where the DEGs were considered significantly enriched at *p* < 0.05.

### Gene expression analysis by quantitative PCR

The researchers used Primer 3.0 for designing the primers for the DEGs in response to the cold stress (Table 1). Primer synthesis was done by Majorbio Biomedical Technology Co., Ltd. (Shanghai, China). The researchers used the Bestar^TM^ qPCR RT Kit for the reverse transcription of the RNA into cDNA and further used this cDNA as a template for qPCR. They used the ChamQ SYBR Color qPCR Master Mix (2×) reagent for qPCR detection using the ABI7300 fluorescence quantitative PCR (qRT-PCR) instrument. Each sample was quantified using three parallel PCR devices to reduce the error. Furthermore, they applied the 2^−△△Ct^ technique for calculating the relative expression of every candidate gene and normalized the gene expression to that of the 18S rRNA gene.

**TABLE 1:** Primer sequences used for RT-qPCR.

Genes	Primer sequence (5′-3′)		Product length (bp)	Amplification (%)
*18sRrna*	F	TGT​CCG​GTA​ACC​TTC​GTG​TG	240	95.50
	R	AAT​GCT​TTC​GCT​TTC​GTC​CG		
*ATP synthase F(0)*	F	AGGTCAAAACCGAGGCAC	264	92.73
	R	CAGACAAAGCAAATCCCA		
*ATP synthase F1 subunit alpha*	F	GTGTCGTTGATGCTTTAG	280	96.79
	R	AGTTTTACCAGTCTGTCG		
*Acetyl-CoA acetyltransferase 2*	F	CCTTGATCCATCCGTCAT	102	92.30
	R	TTCCTTCCACCAGTCCTC		
*Acyl-CoA desaturase-like*	F	ACATAATGCCAGAAGAGG	236	97.87
	R	GGATGAAATAAGCCACCC		
*Malonyl-CoA decarboxylase*	F	GTGCCTGGTATCTGTATG	220	92.50
	R	GTGATGACTTTGTTGTGC		
*Phosphoenolpyruvate carboxykinase*	F	TGCTAATTTCACATCTCC	272	93.09
	R	ACACTCAACCTTCCAACC		
*IDH1*	F	AGTTTGAGGCTAAGGGGA	280	96.83
	R	GCGATAGGGTTTGTGGAT		
*Arginase-1-like*	F	TCAAAGATAAAAAAACCC	252	97.56
	R	AAAACAGCCTAACACAAG		
*Serine/threonine-protein phosphatase 2A catalytic subunit alpha isoform-like*	F	AGCAGACAGATCACGCAG	110	99.56
	R	ACCACAACAGGTCGCACA		
*Pyrroline-5-carboxylate reductase 1*	F	ATCTCTCCTGCTGCTATT	116	95.09
	R	CCGCTCTTGTTGTTAAAC		
*Inosine triphosphatase*	F	CGAGACTTTGGATGGGAT	290	100.33
	R	TCAGTGTGTTTGGTGCGT		
*Phosphoribosyl pyrophosphate synthetase 1*	F	GTGGCTGCGGTGAGATAA	118	96.32
	R	TGACGGGCATAAGGAAAA		

### Metabolomic analysis and date annotation

For metabolomics analysis, the researchers used different solvents, such as acetonitrile, ammonium hydroxide, ammonium acetate, methanol, and 2-Chloro-L-phenylalanine (CNW Technologies, China), for extracting the metabolites from the collected serum samples. The impurities and insoluble matter were removed through centrifugation or filtration. The samples were subjected to the LC-MS analysis using the Ultra-Performance Liquid Chromatography 1290 (UPLC, Agilent, United States) that was coupled with the ACQUITY UPLC BEH Amide 1.7 μm column (2.1 × 100 mm) (Waters, United States). An optimal mobile phase was used for every run and subjected to a linear gradient system of ammonium acetate (25 mM) + Ammonium hydroxide (25 mM) solution (Mobile phase A) and acetonitrile (Mobile phase B). Every sample was chromatographed at a flow rate of 500 μL/min, based on the following solvent gradient: 0–0.5 min (5% A + 95% B); 0.5–7 min (35% A+ 65% B); 7.5–8 min (40% A+ 60% B); 8–9 min (40% A+ 60% B); 9–9.1 min (5% A+ 95% B); 9.1–12 min (5% A+ 95% B). The researchers set the following ElectroSpray Ionization (ESI) ion source parameters for analyzing the products that were detected during the chromatographic run: Gas Ion Source (GSI) = 60 Psi; Gas II (GSII) = 60 Psi; curtain gas = 35 Psi; source temperature = 650°C; and Ion Spray voltage (IS) = −4000 V (negative ion mode). The researchers used the Proteo Wizard and XCMS software for exacting the raw peaks, identifying the peaks, aligning the peaks, and integrating the peak areas. They considered the metabolite peak that was detected in more than 50% of the samples for further analyses. They also conducted the orthogonal projections to latent structure-discriminant analysis (OPLS-DA) using the R^2^ and Q^2^ for differentiating between the classes in every group before screening for the differentially expressed metabolites. The significantly differentially expressed metabolites in the 2 groups (i.e., VP and WT) were analyzed using the first principal component of the Variable Importance in the Projection (VIP) values (VIP > 1) as well as the Student’s t-test (*p* < 0.05). The significantly differentially expressed were subjected to the KEGG pathway analysis.

### Integrated transcriptomics and metabolomics analysis

In the present study, the researchers subjected the metabolomic and the transcriptomic data to the integrated KEGG pathway analysis. They determined the Pearson Correlation Coefficient (PCC) and relevant *p*-values for assessing the relationship between the DEGs and differentially expressed metabolites. The samples with PCC > 0.8 and a PCCP < 0.05, were regarded as significant.

### Intestinal microbiota analysis

The researchers extracted the microbial genomic DNA from the yellow catfish intestinal samples with the help of the FastDNA® Spin Kit for Soil (MP Biomedicals, United States) based on the manufacturer’s instructions. They tested the extracted DNA by electrophoresing the samples on the 1% (w/v) agarose gel and estimated the concentration and the purity of the DNA in the samples using the NanoDrop 2000 UV-vis spectrophotometer (Thermo Scientific, Wilmington, United States). Then, they amplified the hypervariable V3-V4 region of the bacterial 16S rRNA gene with the aid of the following primers: 338F (5′-ACT​CCT​ACG​GGA​GGC​AGC​AG-3′) and 806R (5′-GGACTACHVGGGTWTCTAAT-3′) on the GeneAmp® 9700 PCR thermocycler (ABI, CA, United States). The following PCR conditions were set: initial DNA denaturation at 95°C for 3 min, followed by 29 cycles of denaturing at 95°C for 30 s, strand annealing at 55°C for 30 s, extension at 72°C for 45 s, and further extension at 72°C for 10 min, and finally, holding the reaction sample at 4°C. The PCR mixture was as follows: template DNA (10 ng) + 5 × TransStart FastPfu buffer (4 μL) + 2.5 mM dNTPs (2 μL) + forward primer (5 μM, 0.8 μL) + reverse primer (5 μM, 0.8 μL), TransStart FastPfu DNA Polymerase (0.4 μL) + ddH_2_O (12 μL), so that the final sample volume is 20 μL. All PCR reactions were carried out in three sets. The PCR samples were electrophoresed on a 2% (w/v) agarose gel and the products were extracted, purified with the help of an AxyPrep DNA Gel Extraction Kit (Axygen Biosciences, Union City, CA, United States) based on the manufacturer’s instructions, and quantified using the Quantus™ Fluorometer (Promega, United States).

Thereafter, all purified amplicons were pooled in the equimolar, and paired-end sequences were sequenced on the MiSeq PE300 platform/NovaSeq PE250 platform (Illumina, San Diego, United States) based on the standard protocols developed by Majorbio Biomedical Technology Co., Ltd (Shanghai, China). Then, the researchers deposited all raw reads into the NCBI Sequence Read Archive (SRA) database. (Accession Number: SRPPRJNA853914).

The researchers demultiplexed the raw 16S rRNA gene sequencing reads, quality-filtered them using the Fastp ver. 0.20.0 (Chen et al., 2018), and combined them using FLASH ver. 1.2.7 (Magoč and Salzberg, 2011) based on the below criteria: 1) 300 bp reads were truncated at any of the sites if they showed an average quality score <20 when analyzed by the 50 bp sliding window. The truncated reads that were <50 bp or consisted of ambiguous characters were eliminated; 2) The overlapping sequences with >10 bp were assembled based on their overlapped sequence. The researchers set a maximal mismatch ratio of 0.2 for the overlap area, The reads that cannot be assembled were eliminated; 3) All samples were differentiated based on the primers and barcodes. The direction of the sequence was adjusted, and the exact barcode match and 2 nucleotide mismatches during primer matching were determined.

Then, they clustered the operational taxonomic units (OTUs) having a 97% similarity cutoff value (Stackebrandt and Goebel, 1994; Edgar, 2013) using the UPARSE ver. 7.1 (Edgar, 2013). They identified and eliminated all chimeric sequences. They analyzed the taxonomy of every OTU representative sequence using the RDP Classifier ver. 2.2 (Wang et al., 2007) and compared it against the 16S rRNA database (e.g., Silva v138) with a confidence threshold value of 0.7.

### Statistical analysis

All biochemical experiments were conducted in triplicates. The researchers expressed the serum biochemical data as the mean value ± standard error. They conducted the student’s t-test for comparing the average values. All statistical analyses were carried out using the SPSS ver. 17.0 software (SPSS Inc, Chicago, IL, United States). Results with *p* < 0.05 were considered to be statistically significant.

## Results

### Serum biochemical indices

The samples collected from the fish that were subjected to cold stress showed a significant decrease in their glucose and triglycerides levels (*p* < 0.05). On the other hand, their cholesterol and low-density lipoprotein levels showed a significant increase (*p* < 0.05) (Table 2).

**TABLE 2:** Serum biochemical index of G0 group and T0 group (*n* = 3).

Items	G0	T0
Albumin ALB/(g/L)	10.73 ± 0.23	10.73 ± 0.20
Globulin GLOB/(g/L)	17.40 ± 0.64	15.80 ± 0.40
Total protein TP/(g/L)	28.13 ± 0.85	26.53 ± 0.23
Glucose GLU/(mmol/L)	3.85 ± 0.16^b^	3.09 ± 0.21^a^
Triglycerides TG/(mmol/L)	4.82 ± 0.35^b^	3.04 ± 0.27^a^
Low density lipoprotein LDL-C/(mmol/L)	0.24 ± 0.01^a^	0.44 ± 0.04^b^
High-density lipoprotein HDL-C/(mmol/L)	0.21 ± 0.02	0.25 ± 0.02
Cholesterol CHOL/(mmol/L)	3.48 ± 0.15^a^	4.05 ± 0.08^b^

In the same line, values with no letter or the same letter superscripts mean no significant difference (*p* > 0.05), while different small letter superscripts mean significant differences (*p* > 0.05).

### RNA-seq analysis

The researchers obtained a total of 148,078,020 and 139,632,088 raw reads from the samples collected from the 2 groups. After eliminating the low-quality, adapter, poly-N sequences, 146540750 and 138349654 clean reads were obtained. All the samples showed a Q20, Q30, and GC content higher than 98%, 93%, and 46%, respectively. Then, the researchers aligned the high-quality clean reads with the yellow catfish genome. The alignment rate was over 66%, indicating that the sequencing data that was obtained was of a higher quality and could be effectively used for subsequent analysis (Table 3). Samples G0-1, G0-2, and G0-3 belonged to the G0 group, and samples T0-1, T0-2, and T0-3 belonged to the T0 group.

**TABLE 3:** Sequencing data quality and sequence alignment for all samples.

Samples	Raw reads	Clean reads	Q20 (%)	Q30 (%)	GC content (%)	Uniquely mapped rate (%)
G0-1	45459690	44959954	98.26	94.8	46.57	66.6
G0-2	52360794	51865120	98.32	95.01	47.02	67.97
G0-3	50257536	49715676	98.25	94.82	48.29	66.24
T0-1	48690202	48264612	98.1	94.4	47.04	68.99
T0-2	42318576	41960680	98.14	94.5	47.05	68.58
T0-3	48623310	48124362	97.91	93.96	47.09	69.02

Samples G0-1, G0-2, G0-3 belonged to G0 group, and samples T0-1, T0-2, T0-3 belonged to T0 group.

### Differentially expressed genes gerived from the RNA-Seq analysis data

**FIGURE 1:**
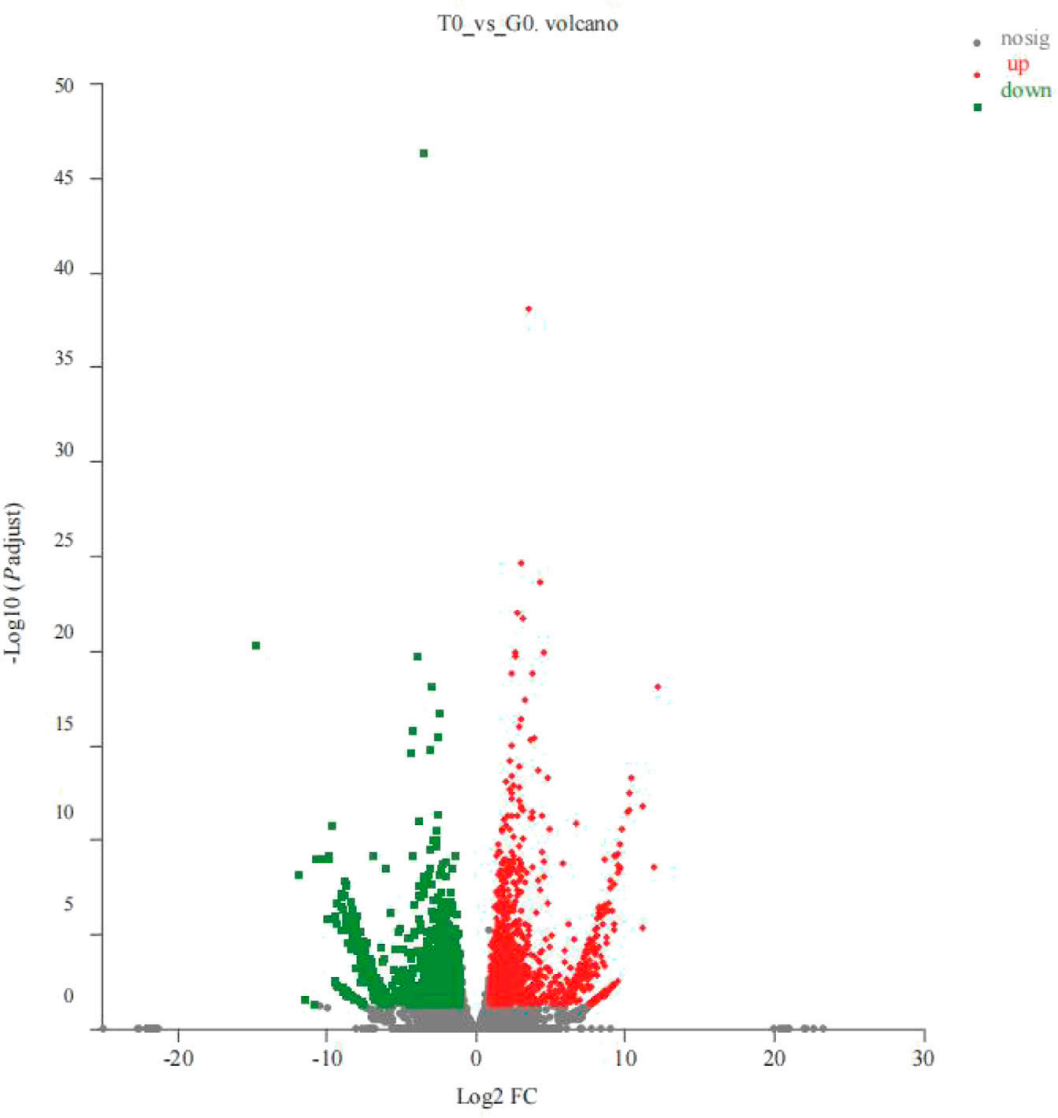
Volcano plots for the DEGs derived from the 2 groups (T0 vs. G0 groups). In the volcano plot described above, the X-axis represented the differences in the gene expressions observed between the 2 groups. The Y-axis depicts the statistical significance of the difference in gene expression levels. The red dots denote the significantly upregulated genes, the green dots depict the significantly downregulated genes, and the gray dots describe the insignificant differential gene expression. *P*-adjust < 0.05 (For interpreting the color scheme used in the figure legends, the readers can refer to the web version of the research article).

Based on the TPM, the absolute value of fold change >2 and *P*-adjust < 0.05 were used as a threshold to analyze the expression levels of the same gene in different groups. Thereafter, the researchers noted that 3350 genes, that included the 1783 upregulated and 1567 downregulated genes, from the control and the treatment groups, were significantly and differentially expressed (Figure 1).

### Gene ontology and kyoto encyclopedia of genes and genomes enrichment analysis of the differential unigenes

**FIGURE 2:**
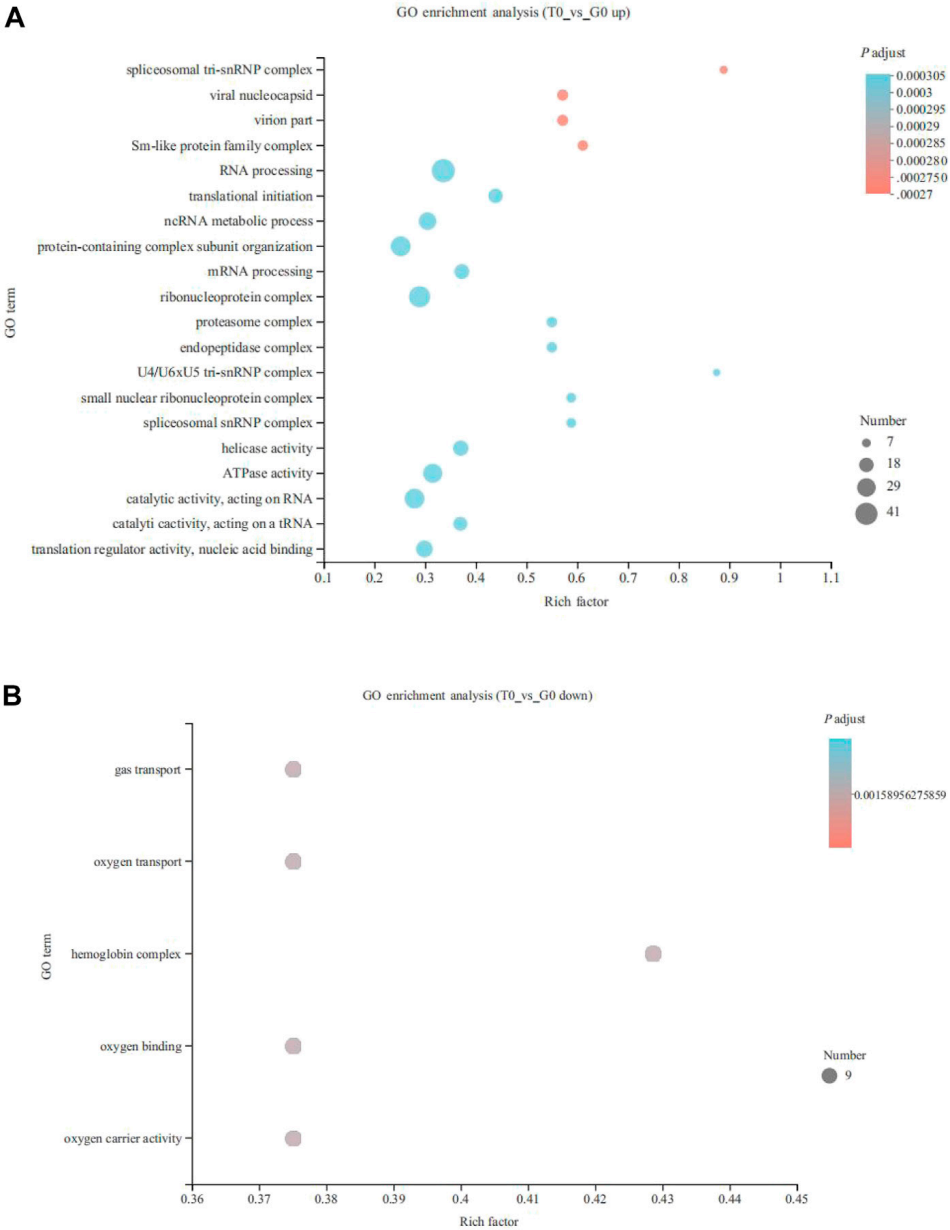
Gene Ontology (GO) enrichment analysis of the DEGs derived from the fish subjected to cold stress. **(A)** upregulated genes. **(B)** downregulated genes.

Unigenes were annotated into 3 primary categories, namely, cellular components, molecular functions, and biological processes. The GO enrichment analysis revealed that 190 upregulated and five downregulated genes annotated significantly to GO terms (*p* < 0.05). The top 20 GO terms associated with the upregulated genes were mainly annotated to RNA processing (41 genes), ribonucleoprotein complex (35 genes), catalytic activity, acting on RNA (31 genes), ncRNA metabolic process (25 genes), and ATPase activity functions (29 genes) (*p* < 0.05) (Figure 2A). While, the five GO terms of downregulated genes were annotated as gas transport (9 genes), oxygen transport (9 genes), hemoglobin complex (9 genes), oxygen binding (9 genes), and oxygen carrier activity (9 genes) (*p* < 0.05) (Figure 2B).

**FIGURE 3:**
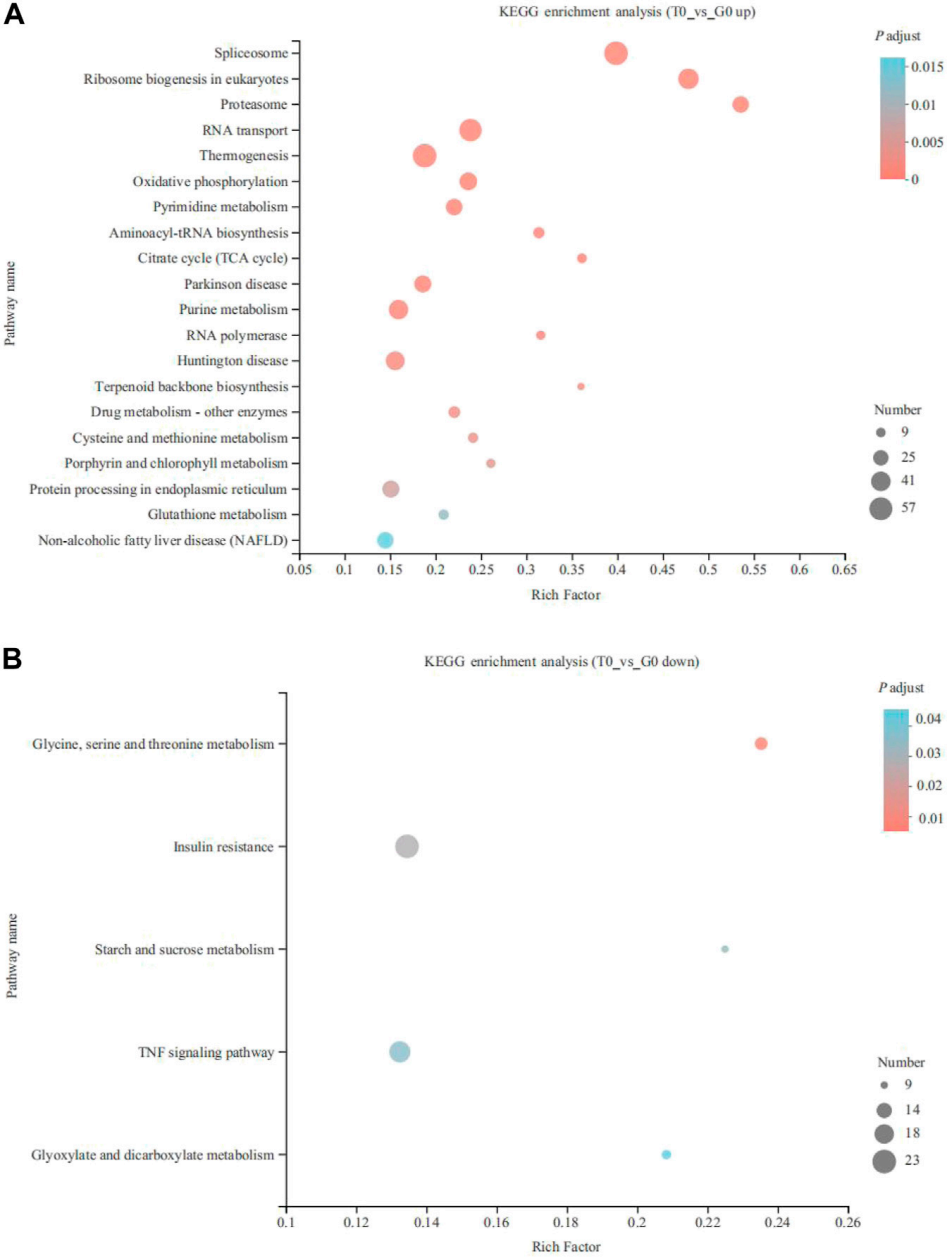
KEGG pathway enrichment analysis of the DEGs derived from the fish subjected to the cold stress. **(A)** upregulated genes. **(B)** downregulated genes.

**FIGURE 4:**
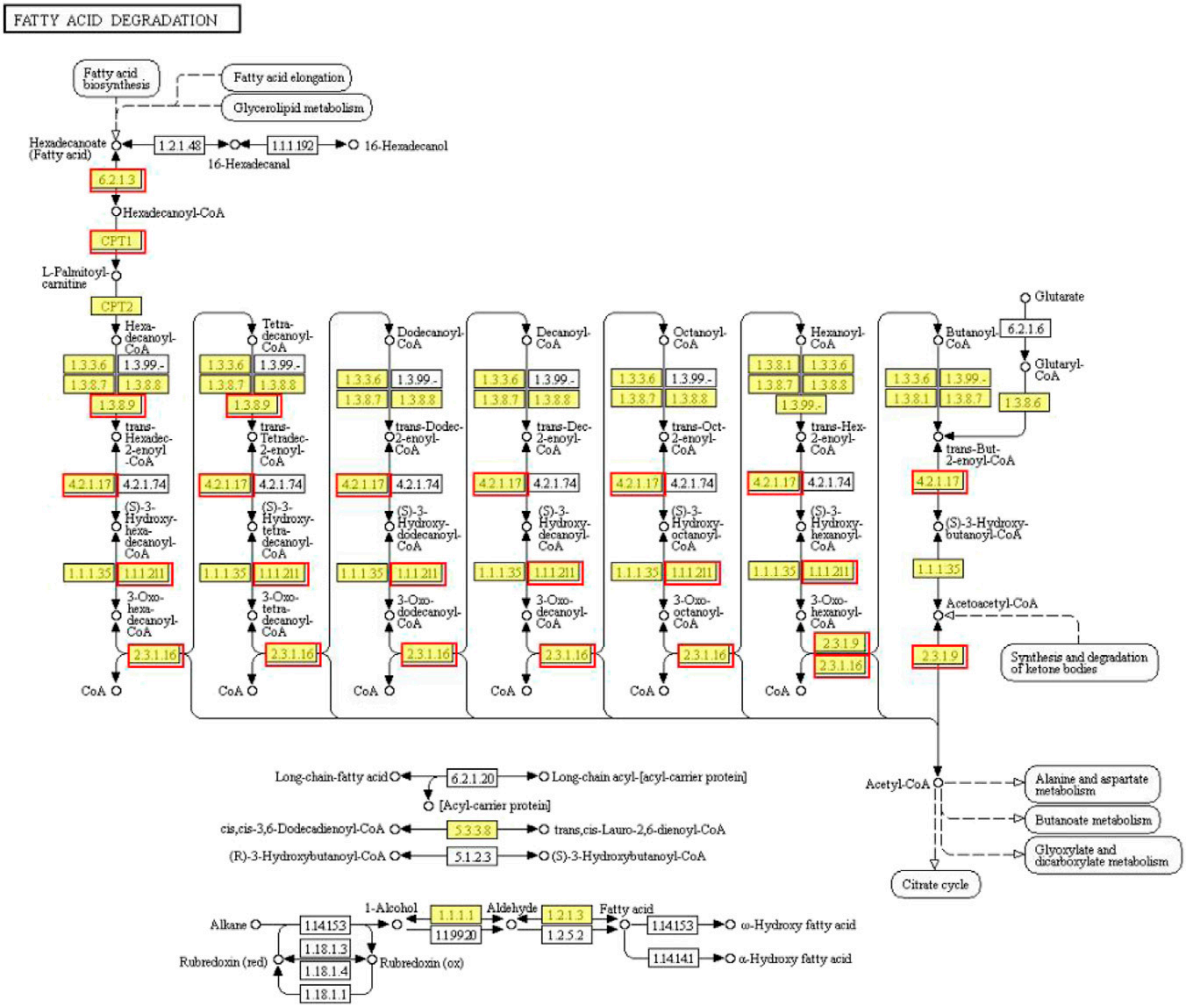
Fatty acid degradation pathway identified in the upregulated genes. The red border represents upregulated genes.

KEGG enrichment analysis indicated that the upregulated and downregulated genes were enriched in 29 and 5 pathways, respectively (*p* < 0.05). The upregulated genes were mainly enriched in thermogenesis (57 genes), spliceosome (57 genes), RNA transport (52 genes), ribosome biogenesis in eukaryotes (44 genes), purine metabolism (40 genes), oxidative phosphorylation (34 genes), protein processing in the endoplasmic reticulum (32 genes), pyrimidine metabolism (30 genes), aminoacyl-tRNA biosynthesis (16 genes) and fatty acid degradation (11 genes) (*p* < 0.05) (Figure 3A). On the other hand, the downregulated genes were enriched in insulin resistance (23 genes), TNF signaling pathway (20 genes), serine, glycine, and threonine metabolism (12 genes), glyoxylate and dicarboxylate metabolism (10 genes), and starch and sucrose metabolism (9 genes) (*p* < 0.05) (Figure 3B). *CPT1* was significantly upregulated and annotated to the fatty acid degradation pathway (Figure 4).

### Validation of the RNA-seq data by qRT-PCR

The relative expressions of 12 DEGs were validated by qRT-PCR (Table 4). The 12 DEGs were as follows: two ATP synthase genes [ATP synthase F(0), ATP synthase F1 subunit alpha], three lipid metabolic genes (acetyl-CoA acetyltransferase 2, acyl-CoA desaturase-like and malonyl-CoA decarboxylase), two carbohydrate metabolism genes (phosphoenolpyruvate carboxykinase, IDH1), two amino acid metabolism genes (arginase-1-like, serine/threonine-protein phosphatase 2A catalytic subunit alpha isoform-like) and three nucleotide metabolism genes (pyrroline-5-carboxylate reductase 1, inosine triphosphatase, phosphoribosyl pyrophosphate synthetase 1). qRT-PCR data were consistent with the RNA-seq data, confirming that RNA-seq data were reliable.

**TABLE 4:** Verification of RNA-Seq by qRT-PCR.

Genes	log2 fold change of RNA-Seq	log2 fold change of qRT-PCR
*ATP synthase F(0)*	2.34	2.38 ± 0.17
*ATP synthase F1 subunit alpha*	2.01	1.86 ± 0.08
*Acetyl-CoA acetyltransferase 2*	2.22	2.67 ± 0.04
*Acyl-CoA desaturase-like*	4.51	3.67 ± 0.18
*Malonyl-CoA decarboxylase*	1.02	1.10 ± 0.64
*Phosphoenolpyruvate carboxykinase*	1.67	2.28 ± 0.24
*IDH1*	2.97	5.71 ± 0.19
*Arginase-1-like*	−1.86	−1.85 ± 0.04
*Serine/threonine-protein phosphatase 2A catalytic subunit alpha isoform-like*	−1.19	−2.00 ± 0.42
*Pyrroline-5-carboxylate reductase 1*	3.84	3.32 ± 0.14
*Inosine triphosphatase*	1.88	2.43 ± 0.36
*Phosphoribosyl pyrophosphate synthetase 1*	1.68	2.79 ± 0.79

### Multivariate statistical analysis and differentially expressed metabolites

**FIGURE 5:**
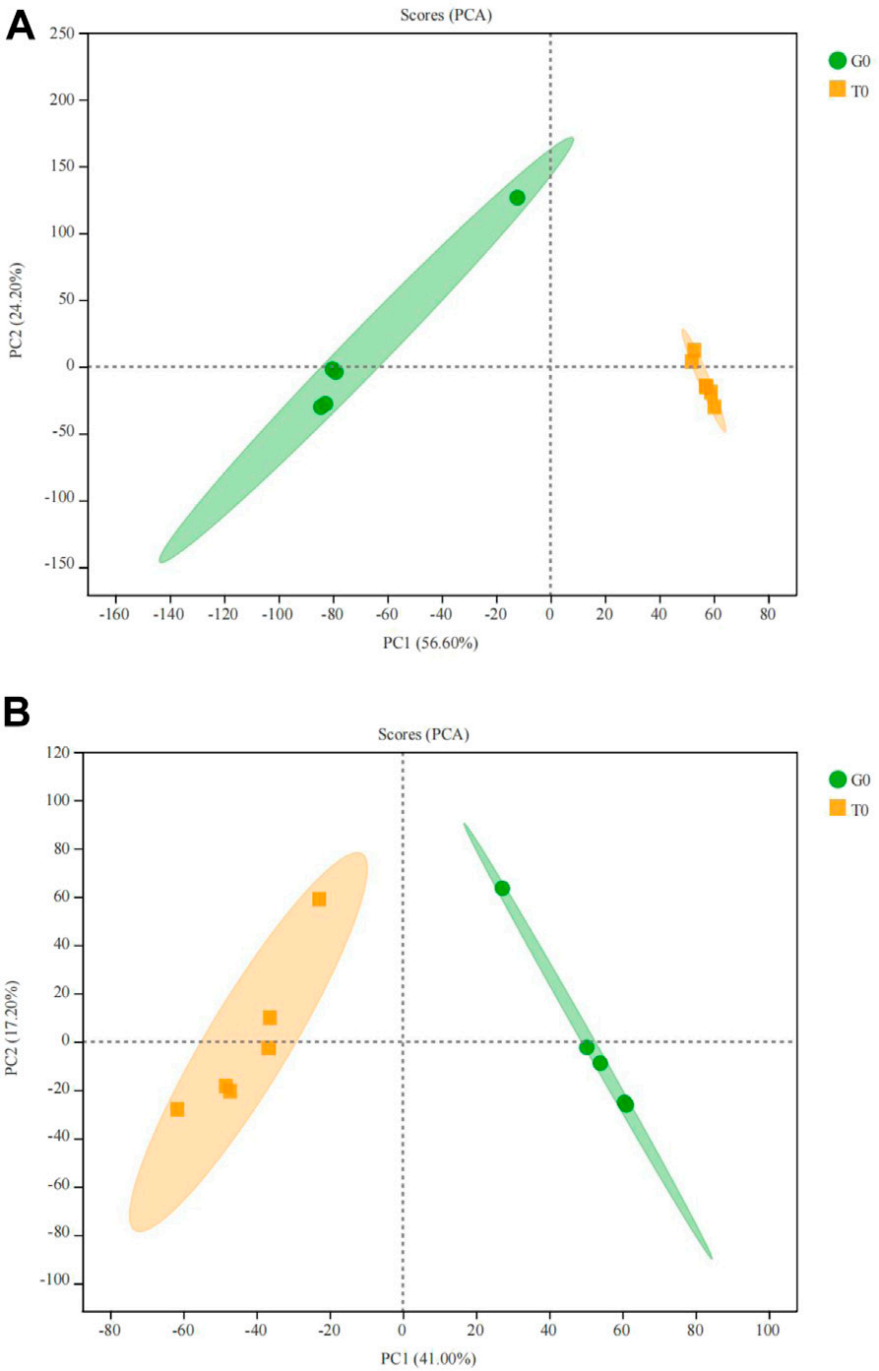
PCA score plot of the serum samples from the two groups. **(A)** positive ion. **(B)** negative ion.

Principal component analysis (PCA) showed that the two groups were completely separated and there existed differences, the R^2^X cum = 0.582 > 0.5, indicating that the PCA model was reliable (Figures 5A,B).

**FIGURE 6:**
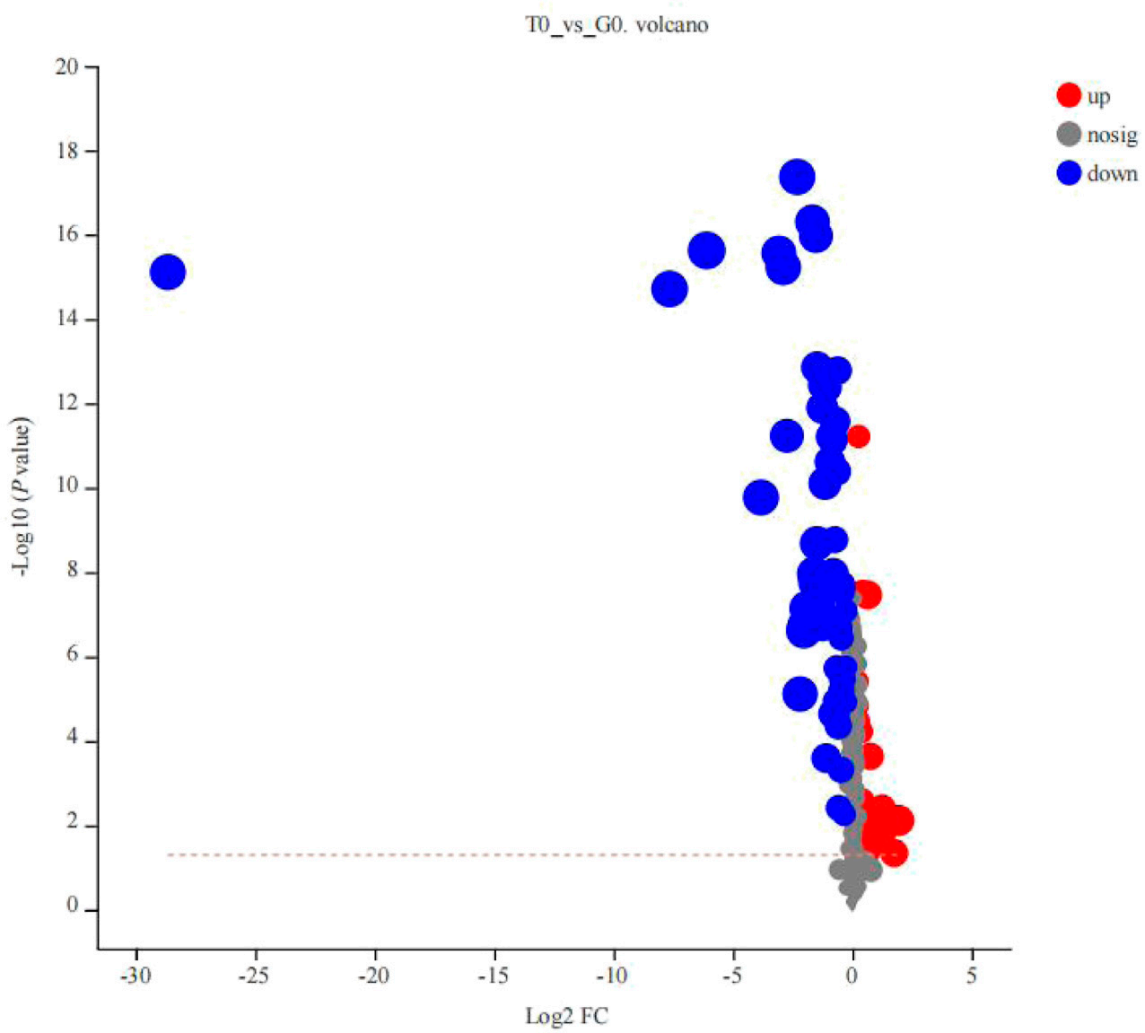
Volcano plot of the differentially expressed metabolites derived from the 2 groups (T0 vs. G0 groups). The X-axis indicated the differences in expression levels between the 2 experimental groups. Y-axis presents the statistical significance of the differential expression of the metabolites. Red dots indicate the significantly upregulated metabolites, blue dots denote the significantly downregulated metabolites, and the gray dots indicate the insignificant differences in the metabolites. *p-value* < 0.05 (For interpreting the color scheme used in the figure legends, the readers can refer to the online version of the research article).

After PCA analysis, the researchers noted that 155 metabolites were differentially expressed in both the groups, i.e., treatment and control groups, wherein 62 were upregulated and 93 were downregulated (Figure 6), with a VIP > 1 and *p* < 0.05. HMDB classification revealed that the upregulated metabolites were mainly lipids and lipid-like molecules (41 species, 68.33%), organic acids and their derivatives (11 species, 18.33%), and organic oxygen compounds (4 species, 6.67%). Downregulated metabolites were mainly lipids and lipid-like molecules (53 species, 66.25%), organic acids and their derivative (8 species, 10.00%), and phenylpropanoids and polyketides (5 species, 6.25%) (Table 5). The 41 species of upregulated lipids and lipid-like metabolites included prenol lipids (16 species, 39.02%), steroids and steroid derivatives (10 species, 24.39%), fatty Acyls (6 species, 14.63%), glycerophospholipids (5 species, 12.20%) and sphingolipids (4 species, 9.76%) (Table 5). The 53 species of downregulated metabolites included fatty acyls (18 species, 33.96%), glycerophospholipids (18 species, 3.96%), prenol lipids (9 species, 16.98%), steroids and steroid derivatives (6 species, 11.32%), glycerolipids (2 species, 3.77%) (Table 5). Moreover, we found that L-glutamine and sphingosine 1-phosphate and 5-hydroxy indole acetic acid were significantly accumulated, while cortisol, sphinganine, LysoPC(22:6(4Z,7Z,10Z,13Z,16Z,19Z)), LysoPC(22:5(4Z,7Z,10Z,13Z,16Z)), LysoPC(20:5(5Z,8Z,11Z,14Z,17Z)), LysoPC(20:4(5Z,8Z,11Z,14Z)), indole acetic acid, and cytidine were significantly consumed (Table 6).

**TABLE 5:** Classification of HMDB compounds for differential metabolites at superclass and class levels.

Superclass	Regulated	Numbers and ratio	Class	Numbers and ratio
Lipids and lipid-like molecules	up	41 (68.33%)	Prenol lipids	16 (39.04%)
			Steroids and steroid derivatives	10 (24.39%)
			Fatty Acyls	6 (14.63%)
			Glycerophospholipids	5 (12.20%)
			Sphingolipids	4 (9.76%)
	down	53 (66.25%)	Fatty Acyls	18 (33.96%)
			Glycerophospholipids	18 (33.96%)
			Prenol lipids	9 (16.98%)
			Steroids and steroid derivatives	6 (11.32%)
			Glycerolipids	2 (3.77%)
Organic acids and derivatives	up	11 (18.33%)		
	down	8 (10.00%)		
Organic oxygen compounds	up	4 (6.67%)		
	down	2 (2.50%)		
Phenylpropanoids and polyketides	up	2 (3.33%)		
	down	5 (6.25%)		
Benzenoids	up	1 (1.67%)		
	down	3 (3.75%)		
Organoheterocyclic compounds	up	1 (1.67%)		
	down	3 (3.75%)		
Organic nitrogen compounds	down	2 (2.50%)		
Organooxygen compounds	down	2 (2.50%)		
Lignans, neolignans and related compounds	down	1 (1.25%)		
Nucleosides, nucleotides, and analogues	down	1 (1.25%)		

**TABLE 6:** Major different metabolites of T0 group vs. G0 group.

ID	Metabolite	Formula	FC(T0/G0)	Regulated
neg_266	L-Glutamine	C_5_H_10_N_2_O_3_	1.16	up
neg_6324	Aspartylphenylalanine	C_13_H_16_N_2_O_5_	1.10	up
pos_1826	Asparaginyl-Arginine	C_10_H_20_N_6_O_4_	1.22	up
pos_8833	Histidinyl-Isoleucine	C_12_H_20_N_4_O_3_	1.20	up
pos_2013	Sphingosine 1-phosphate	C_18_H_38_NO_5_P	7.45	up
neg_5013	5-Hydroxyindoleacetic acid	C_10_H_9_NO_3_	1.14	up
pos_493	Sphinganine	C_18_H_39_NO_2_	0.84	down
neg_549	LysoPC(22:6(4Z,7Z,10Z,13Z,16Z,19Z))	C_30_H_50_NO_7_P	0.97	down
neg_541	LysoPC(20:5(5Z,8Z,11Z,14Z,17Z))	C_28_H_48_NO_7_P	0.95	down
neg_576	LysoPC(22:5(4Z,7Z,10Z,13Z,16Z))	C_30_H_52_NO_7_P	0.95	down
neg_556	LysoPC(20:4(5Z,8Z,11Z,14Z))	C_28_H_50_NO_7_P	0.96	down
pos_293	Cytidine	C_9_H_13_N_3_O_5_	0.84	down
neg_6080	Indolelactic acid	C_11_H_11_NO_3_	0.76	down
neg_436	Cortisol	C_21_H_30_O_5_	0.94	down

### Kyoto encyclopedia of genes and genomes pathways associated with the differentially expressed metabolites

**FIGURE 7:**
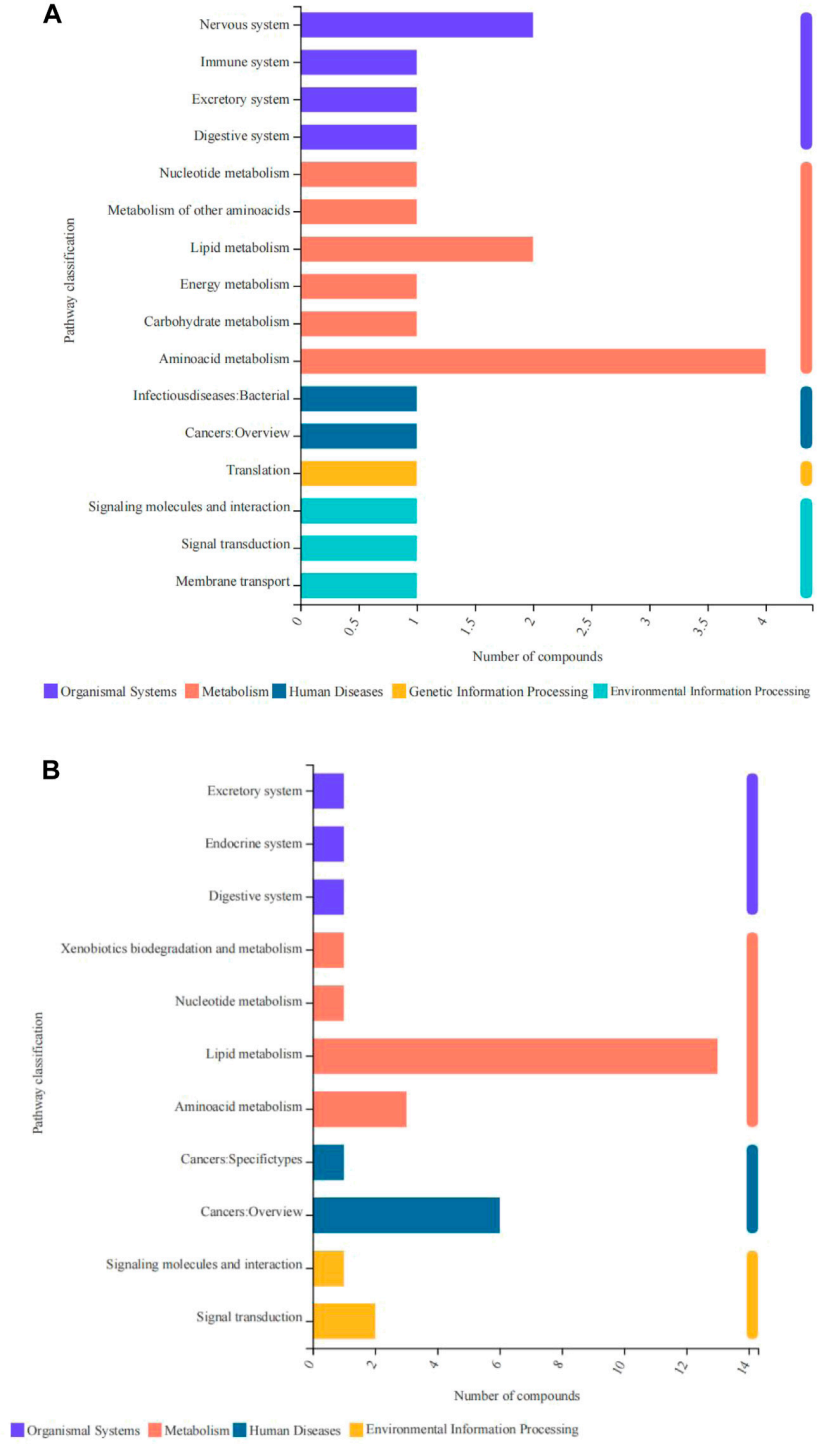
Pathway classification of differentially expressed metabolites derived from the fish subjected to cold stress. **(A)** upregulated metabolites. **(B)** downregulated metabolites.

In this study, differentially expressed metabolites were mainly related to five primary processes, namely, human diseases, metabolism, organismal systems, environmental information processing, and genetic information processing. The upregulated metabolites were mainly associated with energy metabolism, amino acid metabolism, lipid metabolism, nervous system, membrane transport, and nucleotide metabolism (Figure 7A). The downregulated metabolites were mainly related to amino acid metabolism, lipid metabolism, cancers, and signal transduction (Figure 7B).

### Kyoto encyclopedia of genes and genomes pathway enrichment analysis of differentially expressed metabolites

**FIGURE 8:**
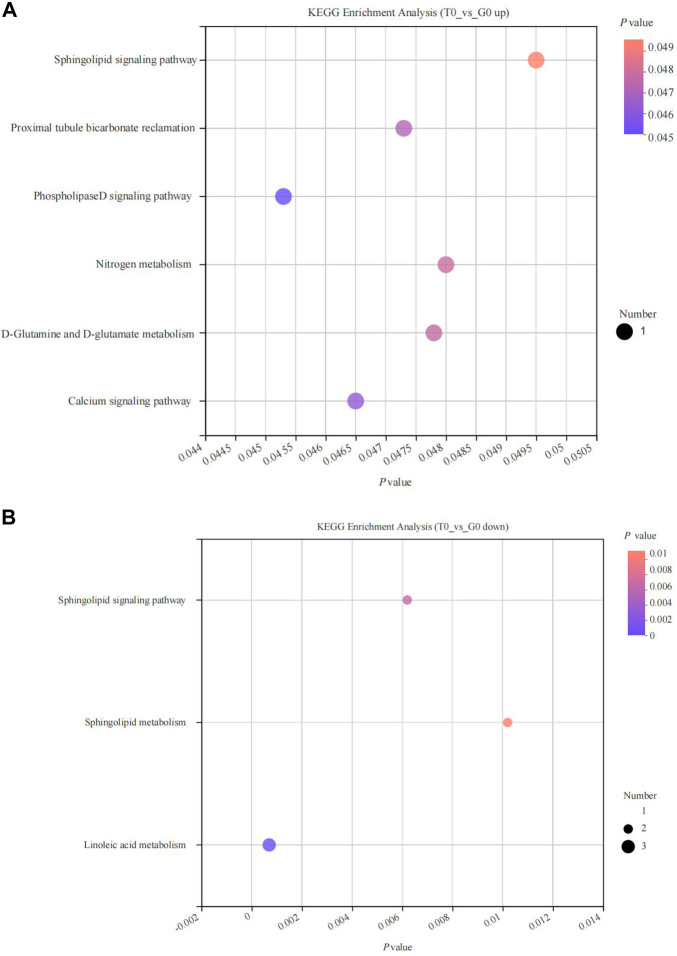
KEGG pathway enrichment analyses of differentially expressed metabolites derived from the fish subjected to cold stress. **(A)** upregulated metabolites. **(B)** downregulated metabolites.

KEGG enrichment analysis revealed that the upregulated and the downregulated metabolites were significantly enriched in six and three pathways, respectively (*p* < 0.05). The upregulated metabolites were enriched in the phospholipase D signaling pathway, calcium signaling pathway, D-glutamine, and D-glutamate metabolism, proximal tubule bicarbonate reclamation, nitrogen metabolism, sphingolipid signaling pathways (Figure 8A), while the downregulated metabolites were significantly enriched in the sphingolipid signaling pathway, sphingolipid metabolism, linoleic acid metabolism pathways (Figure 8B).

### Integrated analysis of transcriptome and metabolome

**FIGURE 9:**
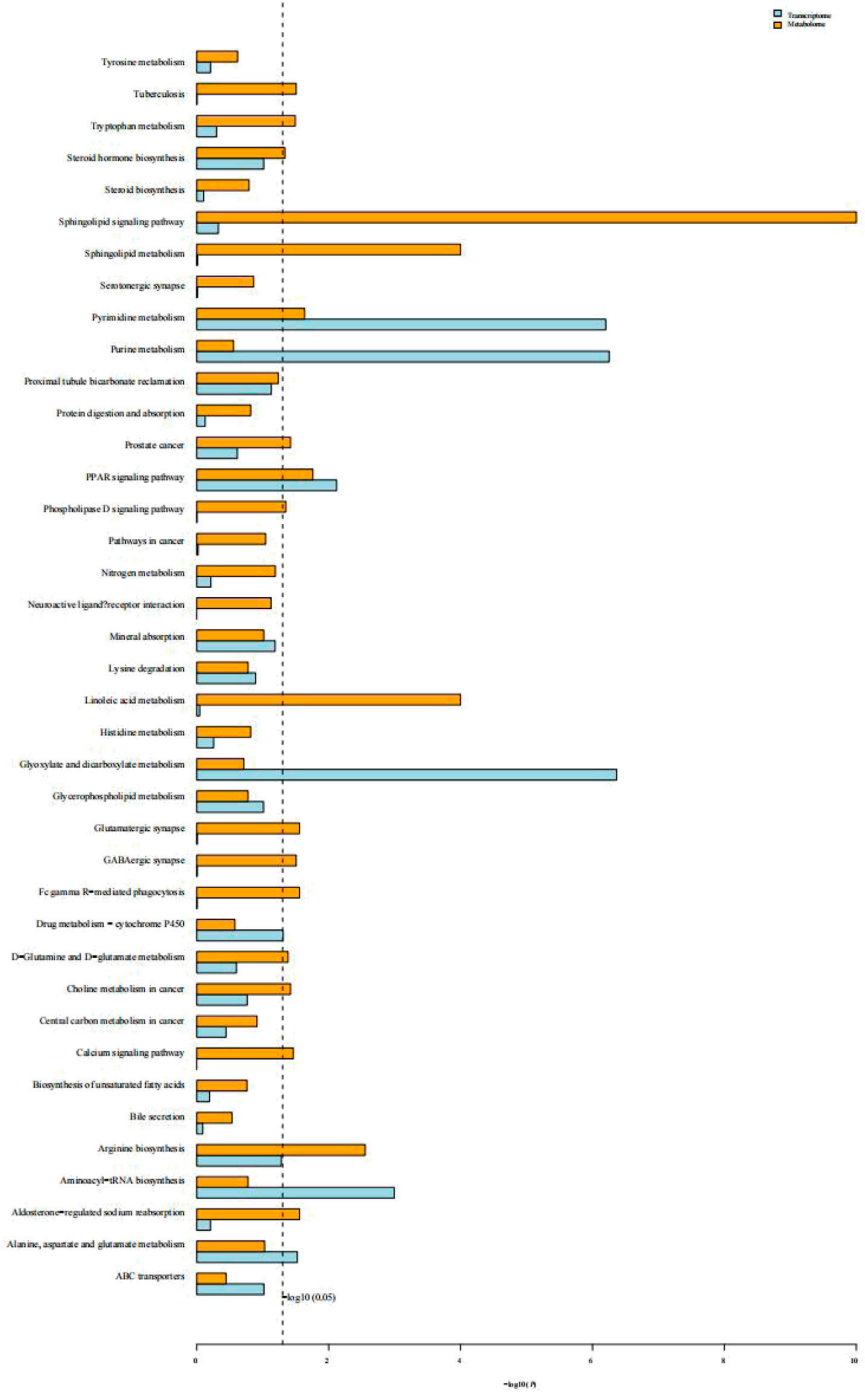
Histogram of the KEGG enrichment analysis of DEGs and differentially expressed metabolites. Blue depicts the transcriptome and yellow depicts the metabolome. The ordinate text indicates the pathway involved. The horizontal axis represents the significance of pathway enrichment, i.e., *p-value* and log *P*. The longer the column, the more significant the association of a biological pathway to the samples tested.

KEGG enrichment analysis of transcriptomic and metabolomics data revealed that both DEGs and the differentially expressed metabolites in response to cold stress were associated with the PPAR signaling pathway, pyrimidine and purine metabolism, glycerophospholipid metabolism, alanine, aspartate, and glutamate metabolism, arginine biosynthesis, aminoacyl-tRNA biosynthesis, and mineral absorption (Figure 9).

### Intestinal microbiota composition

**FIGURE 10:**
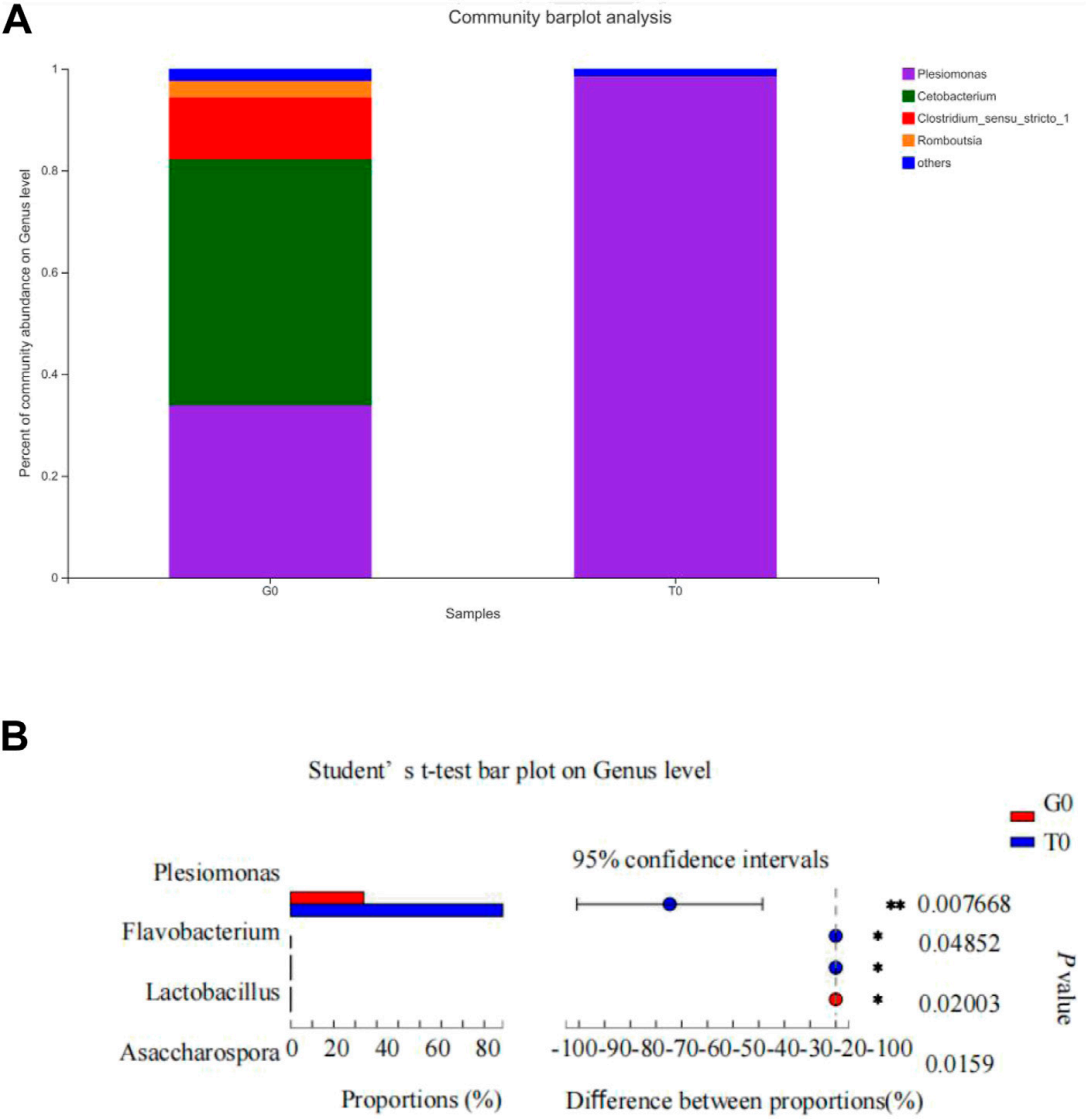
Intestinal microbiota composition. **(A)** Relative abundance of the intestinal microbiota at the genus level. **(B)** Differences in the relative abundance of the intestinal microflora at the genus level.

Community heatmap analysis indicated that the relative abundance of the intestinal microbiome in the fish decreased significantly in response to cold stress. Under non-stressful conditions, the intestinal microbiota was abundant in *Cetobacterium*, *Plesiomonas*, *Clostridium,* and *Romboutsia*, while after cold stress, it was predominant in *Plesiomonas* (Figure 10A). The abundance in the treatment group was observed to be significantly higher compared to that noted in the control group (Figure 10B).

## Discussion

### RNA and protein processing was upregulated in response to cold stress

RNA processing refers to the modification of the original RNA product by deleting some nucleotides and adding some nucleotides not encoded by genes. It is the process of converting transcription products into mature RNA molecules and is crucial for the subcellular localization, translation, and stability of transcripts (Hocine et al., 2010). ncRNAs include miRNA, lncRNA, and circRNA, among others, and they are seen to play a significant role in several biological activities. Here, the researchers noted that the unigene function of RNA processing, catalytic activity (acting on RNA), and ncRNA metabolic processes were significantly upregulated. Similar results have also been obtained in zebrafish (*Danio rerio*) (Li et al., 2012), *Cyprinus carpio* (Gracey et al., 2004), and yellow drum (*Nibeaal biflflora*) (Xu et al., 2018), when these species were subjected to cold stress. Therefore, the establishment of a low-temperature adaptive phenotype requires alteration of RNA splicing, modification, and degradation to prevent the formation of RNA secondary structure under low-temperature conditions (Li et al., 2012). Ribonucleoproteins are the factory for protein synthesis, and the upregulated expression of ribosomal biogenic genes may be a compensatory response to promote protein synthesis at low temperatures (Li et al., 2012), and has been observed in zebrafish (Li et al., 2012), as well as in yellow catfish in our study.

### Aerobic respiration was downregulated in response to cold stress

Temperature affects many biological processes, including aerobic respiration. Several species, including *Miichthys miiuy* (Zheng et al., 2008), *Triakis Semifasciata* (Miklos et al., 2003), gilthead sea bream (*Sparus aurata*) (Ibarz et al., 2003) *Ptychobarbus kaznakovi* (Ke et al., 2019), Atlantic salmon (*Salmo salar*) (Madaro et al., 2018), mahseer (*Tor tambroides*) (Das et al., 2018) have been shown to exhibit significantly decreased respiration rate with a decrease in temperature. In this study, we demonstrated that gas transport, oxygen transport, hemoglobin complex, oxygen binding, and oxygen carrier activity of unigenes decreased under cold stress. Therefore, like other aquatic animals, yellow catfish also show reduced oxygen consumption to adapt to a low-temperature environment.

### Thermogenesis and oxidative phosphorylation were induced in response to cold stress

It has been reported that adaptive thermogenesis is a very important survival mechanism that is adopted by homeotherms for adapting to the ambient temperatures that fall below the thermoneutrality (Silva, 2003). Researchers noted enhanced mitochondrial activity in the brown adipose tissue, along with an increase in the electron transport activity, the β-oxidation process of fatty acids, and the UnCoupling Protein 1 (UCP1) expression, for heat generation during the cold stress (Cannon and Nedergaard, 2004; Blondin et al., 2014; Blondin et al., 2017). Although we did not observe brown adipose tissue in fish, previous studies have shown that fish express many UCP molecules that can be induced by low temperature in adipose tissue (Jastroch et al., 2005; Coulibaly et al., 2006; Jastroch et al., 2007). We did confirm that the thermogenesis pathways were significantly enriched and upregulated. Oxidative phosphorylation or mitochondrial respiratory chain helps in controlling the energy generation and ATP synthesis in eukaryotic species (Saraste, 1999), and is hypothesized to be controlled by the tissue metabolic stress for maintaining energy metabolism homeostasis (Phillips et al., 2012). They play a vital role in biological growth, metabolism, and environmental adaptation. We found that oxidative phosphorylation was significantly activated as a response to the cold stress even in other aquatic animals, like the yellow croaker (*Larimichthy crocea*) (Qian and Xue, 2016), shrimp (Fan et al., 2019), and *Macrobrachium nipponense* (Jin et al., 2020). Energy expenditure is needed for poikilotherms to maintain metabolic homeostasis under cold stress. Thermogenesis and oxidative phosphorylation were the main biological processes that generated energy for the purpose of maintaining metabolic homeostasis in yellow catfish exposed to cold stress.

### Amino acid metabolism was induced in response to cold stress

Free amino acids in ectothermic animals enable several functions, including protein synthesis, regulating energy metabolism, growth, metamorphosis, and osmotic pressure balance (Karanova and Andreev, 2010). Environmental temperature influences the pool of free amino acids and changes free amino acid composition as temperature declines to negative or near-zero values (Hazel and Prosser, 1974). There exists a positive correlation between the increase in some free amino acids and water temperature decline, and this phenomenon is considered a protective response to adapt to cold stress (Karanova, 2006). Under cold stress, many changes in amino acid metabolism-associated genes or amino acid levels were observed in yellow croaker (*Larimichthy crocea*) (Qian and Xue, 2016), orange-spotted groupers (*Epinephelus coioides*) (Sun et al., 2019), red claw crayfish (*Cherax quadricarinatus*) (Wu et al., 2019), and the kuruma shrimp (*Marsupenaeus japonicus*) (Ren et al., 2020). Similarly, we found that the unigenes of aspartate, alanine, and glutamate metabolism, D-glutamate and D-glutamine metabolism, arginine biosynthesis, and aminoacyl-tRNA biosynthesis were significantly upregulated, as determined by RNA-seq data, indicating that cold stress stimulated amino acid metabolism (Ren et al., 2020). Glutamate and glutamine are the most abundant free amino acids and act as the major metabolic fuel for fish tissues (Li et al., 2020). Previous studies have shown that glutamate levels in kuruma shrimp (Ren et al., 2020) and *Drosophila melanogaster* (MacMillan et al., 2016) were significantly increased in response to cold stress. Similarly, we found that the metabolites L-glutamine, aspartyl-phenylalanine, asparaginyl-arginine, and histidinyl-isoleucine were significantly accumulated in yellow fish under cold stress. In some regards, amino acids are considered an important energy source in fish (Ghisaura et al., 2019). Therefore, we deduced that an artificial supply of certain amino acids, such as L-glutamine, which acts as a metabolic precursor of glutathione, proline, and arginine, may protect yellow catfish against cold stress. The supplementation of alanyl-glutamine dipeptide and VE in the diet has promoted the growth and the antioxidation mechanism in the genetically enhanced farmed tilapia (*Oreochromis niloticus*) juveniles at low temperatures (Xu et al., 2020).

### Lipid metabolism was induced in response to cold stress

Lipid metabolism plays a key role in converting the nutrients into metabolic intermediates that are used further for storing energy, membrane biosynthesis, signaling molecule production, and resistance to cold stress (Hsieh et al., 2003a; Ibarz et al., 2005; Röhrig and Schulze, 2016; Sacristán et al., 2016; DeBose-Boyd, 2018). Mitochondrial β-oxidation of fatty acids produces excessive energy for physical activity and also protects the poikilotherms from cold stress. In our study, KEGG enrichment analysis revealed that the fatty acid degradation pathway and the expression of CPT1 were both upregulated. CPT1 is a rate-limiting step of the carnitine palmitoyl transferase system, which loads the long-chain fatty acids into the mitochondria for oxidative decomposition (Morash et al., 2008). Correspondingly, the HMDB classification revealed that fatty acyls were downregulated and the levels of unsaturated fatty acids such as lysoPC(22:5(4Z,7Z,10Z,13Z,16Z)), lysoPC(22:6(4Z,7Z,10Z,13Z,16Z,19Z)), lysoPC(20:4(5Z,8Z,11Z,14Z)), and lysoPC(20:5(5Z,8Z,11Z,14Z,17Z)) were also decreased in our study. Similarly, in an earlier study, the researchers noted that the CPT1 expression level was increased in the orange-spotted groupers (*Epinephelus coioides*) subjected to cold stress, which indicated that the β-oxidation of long-chain fatty acids was enhanced to provide energy (Sun et al., 2019). However, CPT1 expression in the *Cherax quadricarinatus* was decreased under cold stress possibly due to prolonged starvation or the inhibition of long-chain unsaturated fatty decomposition owing to the membrane phase transition theory (Wu et al., 2019).

Glycerophospholipids and sphingolipids make up the main component of the lipid matrix of biological membranes. Glycerophospholipid metabolism and sphingolipid metabolism are two membrane fluidity-related pathways. Sphingolipid metabolism is significantly upregulated in zebrafish under cold stress (Wang et al., 2014), while it was found to be significantly downregulated in our study. Consequently, the metabolites of sphinganine and dihydroceramide were significantly decreased. A similar result was found in the kuruma shrimp (*Marsupenaeus japonicus*) (Ren et al., 2020). Poikilotherms have different mechanisms to adapt to cold stress such as by altering the cell membrane through increased sphingolipid metabolism, steroid biosynthesis, cholesterol transport, and/or increased unsaturated fatty acid accumulation (Gracey et al., 2004; Hu et al., 2014; Wang et al., 2014; Fan et al., 2019; Meng et al., 2019). In our study, serum cholesterol and low-density lipoprotein levels were significantly increased, meanwhile metabolomics revealed the prenol lipids, steroids, and steroid derivatives were significantly increased. An increase in total cholesterol has been reported in *Lateolabrax maculatus* to serve as an important energy material under cold stress (Wang et al., 2022). Thus, we postulated that the lipid molecules like prenol lipids, steroids, and steroidal derivatives help in maintaining the cell membrane stability, thereby acting as the source of energy for the yellow catfish under cold stress.

### Carbohydrate metabolism was increased in response to cold stress

Glucose is an energy substrate and is considered the preferred energy source for consumption in the early stages of cold stress (Lucas, 1996; Lermen et al., 2004). Usually, the plasma glucose content can be increased by enhancing the glycogenolysis and gluconeogenesis pathways, which increases the energy supply (Woo, 1990; Sun et al., 1995). Furthermore, the sucrose and starch metabolism, galactose metabolism, and the pentose and glucuronate interconversions are activated to convert other carbohydrates into glucose to compensate for the energy deficit after cold exposure (Jiao et al., 2020). We found that glucose was significantly increasingly consumed after cold stress. Similarly, plasma glucose levels in pacific white shrimp (Wang et al., 2019) and mucus glucose in gilthead sea bream (Sanahuja et al., 2019) were also reduced in response to cold stress. On the gene level, we found the carbohydrate metabolism activity was decreased since the starch and sucrose metabolism, as well as the glyoxylate and dicarboxylate metabolism, were significantly downregulated. Similarly, in shrimp, under cold stress, the carbohydrate metabolism was downregulated at 13°C, suggesting that carbohydrate is probably used as a source of energy during the initial cold stress stages, however, not long-term (Fan et al., 2019). The metabonomic analysis in our study showed the organic acids and derivative was the second metabolites inferior to the lipids and lipid-like molecules.Likewise, studies in milkfish (*Chanos chanos*) also confirmed that lipids and lipid-like molecules could be used as a better energy source compared to glucose to maintain the normal metabolic functions for a long stage of cold stress (Hsieh et al., 2003a).

Insulin resistance reflects decreased glucose metabolism in cells and causes hyperglycemia, hyperinsulinemia, hyperlipidemia, and other abnormalities (Kampoli et al., 2011; Sinaiko and Caprio, 2012). Chronic intermittent cold stress has been shown to cause local nerve resistance to insulin in rats. We found the insulin resistance pathway to be downregulated in yellow catfish in response to cold stress. Therefore, we postulate that insulin resistance response in animals may be related to stress tolerance, stress duration, and stress level. Moreover, the observed downregulated insulin resistance may be a protection mechanism to reduce the effect of cold stress in fish as increased glucose was consumed under cold stress.

### Nucleotide metabolism was upregulated in response to cold stress

Nucleotides are necessary for many biological processes, including energy storage and metabolism, DNA and RNA synthesis, activation of phospholipids and carbohydrates, and other metabolic regulators (Voet and Voet, 1995). During cell growth and development, the DNA synthesis is increased via nucleotide metabolism, which further supports the protein synthesis at varying cell-cycle stages (Lane and Fan, 2015). AN increase in the purine metabolism is confirmed to play a vital role in countering cold stress and has been observed in gilthead sea bream (Melis et al., 2017), yellow drum (*Nibea albiflora*) (Xu et al., 2018), and shrimp (Fan et al., 2019). In this study, the results indicated that the expression of several genes that participated in the purine/pyrimidine metabolism was upregulated and cytidine was consumed to resist cold stress.

### Composition of the intestinal microbiota was altered in response to cold stress

Intestinal microbial homeostasis directly affects the digestion, absorption, defense, growth, and development of fish (Schryver and Vadstein, 2014). Increased microbial diversity favors health, while reduced diversity promotes host instability (Al-Masqari et al., 2022). The results showed that the relative abundance of intestinal microbiota was significantly altered due to cold stress. This finding was consistent with those of previous studies on rainbow trout, yellowtail kingfish (*Seriola lalandi*), and *Apostichopus japonicus* (Rong, 2012; Soriano et al., 2018; Cruz-Flores et al., 2022b). In addition, in other freshwater fish like milkfish, *Chanos chanos* (Hassenrück et al., 2020), and Tasmanian Atlantic Salmon (Neuman et al., 2016), reduced alpha diversity has been recorded with increased water temperature. Therefore, we can infer that water temperature is a significant factor that affects intestinal microbiota stability and that drastic fluctuations are not conducive to intestinal microbiota stability. In large yellow croakers, however, temperature reduction did not cause significant changes in intestinal microbiota (Lv et al., 2021). *Plesiomonas* is an important pathogenic genus in many aquatic animals and is considered to be the cause of fish enteritis and intestinal diseases (Duan et al., 2022). We found that the relative abundance of *Plesiomonas* increased significantly with decreasing temperature and become the dominant intestinal microbiota, suggesting that temperature changes may increase the susceptibility of yellow catfish to pathogenic bacteria. A similar effect is seen in shrimp exposed to high temperatures, wherein they show increased susceptibility to *Vibrio* infection and reduced survival rate (Al-Masqari et al., 2022).

## Conclusions

In this study, we established that yellow catfish reduced aerobic respiration activity to adapt to cold stress, but activated RNA processing and ribosome biogenesis to solve the problem of RNA secondary structure changes and protein synthesis. Yellow catfish also showed increased thermogenesis and oxidative phosphorylation to provide energy for maintaining metabolic homeostasis. The increase in lipid levels in response to cold stress not only acted as a source of energy but also contributed to maintaining the stability of the cell membrane. Additionally, amino acid metabolism, insulin resistance, and purine and pyrimidine metabolism helped adapt to cold stress. Furthermore, the change in the relative abundance of the intestinal microbiota and significant increase in the *Plesiomonas* abundance owing to cold stress could impair intestinal health.

## Data Availability

The datasets presented in this study can be found in online repositories. The names of the repository/repositories and accession number(s) can be found below: https://www.ncbi.nlm.nih.gov/, PRJNA853914.

## References

[B1] Al-Masqari Z. A. Guo H. P. Wang R. Y. Yan H. Z. Dong P. S. Wang G. S. (2022). Effects of high temperature on water quality, growth performance, enzyme activity and the gut bacterial community of shrimp (*Litopenaeus vannamei*). Aquac. Res. 53, 3283–3296. 10.1111/are.15836

[B2] Blondin D. P. Daoud A. Taylor T. Tingelstad H. C. Bezaire V. Richard D. (2017). Four-week cold acclimation in adult humans shifts uncoupling thermogenesis from skeletal muscles to brown adipose tissue. J. Physiol. 595 (6), 2099–2113. 10.1113/JP273395 28025824 PMC5350439

[B3] Blondin D. P. Labbe S. M. Tingelstad H. C. Noll C. Kunach M. Phoenix S. (2014). Increased brown adipose tissue oxidative capacity in cold-acclimated humans. J. Clin. Endocrinol. Metab. 99 (3), E438–E446. 10.1210/jc.2013-3901 24423363 PMC4213359

[B4] Cannon B. Nedergaard J. (2004). Brown adipose tissue: Function and physiological significance. Physiol. Rev. 84 (1), 277–359. 10.1152/physrev.00015.2003 14715917

[B5] Chen S. F. Zhou Y. Q. Chen Y. R. Gu J. (2018). Fastp: an ultra-fast all-in-one FASTQ preprocessor. Bioinformatics 34 (17), 884–890. 10.1093/bioinformatics/bty560 30423086 PMC6129281

[B6] Cheng S. Y. Chen C. S. Chen J. C. (2013). Salinity and temperature tolerance of brown marbled grouper *Epinephelus fuscoguttatus* . Fish. Physiol. Biochem. 39 (2), 277–286. 10.1007/s10695-012-9698-x 22869056

[B7] Coulibaly I. Gahr S. A. Yao J. Rexroad C. E. (2006). Embryonic expression of UCP2 in rainbow trout (*Oncorhynchus mykiss*). Fish. Physiol. Biochem. 32 (3), 249–253. 10.1007/s10695-006-9101-x

[B8] Cruz-Flores R. López-Carvallo J. A. Cáceres-Martínez J. Dhar A. K. (2022a). Microbiome analysis from formalin-fixed paraffin-embedded tissues: Current challenges and future perspectives. J. Microbiol. Methods 196, 106476. 10.1016/j.mimet.2022.106476 35490989

[B9] Cruz-Flores R. Rodríguez M. H. Flores J. S. O. G. Dhar A. K. (2022b). Formalin-fixed paraffin-embedded tissues for microbiome analysis in rainbow trout (*Oncorhynchus mykiss*). J. Microbiol. Methods 192, 106389. 10.1016/j.mimet.2021.106389 34863804

[B10] Das S. K. Noor N. M. Kai K. S. Juan Q. Z. Iskandar N. S. M. De M. (2018). Effects of temperature on the growth, gastric emptying time, and oxygen consumption rate of mahseer (*Tor tambroides*) under laboratory conditions. Aquac. Rep. 12, 20–24. 10.1016/j.aqrep.2018.08.004

[B11] DeBose-Boyd R. A. (2018). Significance and regulation of lipid metabolism. Semin. Cell Dev. Biol. 81, 97. 10.1016/j.semcdb.2017.12.003 29246858

[B12] Dietrich M. A. Hliwa P. Adamek M. Steinhagen D. Karol H. Ciereszko A. (2018). Acclimation to cold and warm temperatures is associated with differential expression of male carp blood proteins involved in acute phase and stress responses, and lipid metabolism. Fish. Shellfish Immunol. 76, 305–315. 10.1016/j.fsi.2018.03.018 29544770

[B13] Duan Z. P. Zhang C. Y. Huang L. L. Lan Q. Hu J. Li X. Q. (2022). An evaluation of replacing fish meal with fermented soybean meal in diet of hybrid snakehead (*Channa argus* × *Channa maculata)*: Growth, nutrient utilization, serum biochemical indices, intestinal histology, and microbial community. Aquac. Nutr. 2022, 1–13. 10.1155/2022/2964779

[B14] Edgar R. C. (2013). Uparse: Highly accurate OTU sequences from microbial amplicon reads. Nat. Methods 10 (10), 996–998. 10.1038/NMETH.2604 23955772

[B15] Fan L. F. Wang L. Wang Z. L. (2019). Proteomic characterization of the hepatopancreas in the pacific white shrimp *Litopenaeus vannamei* under cold stress: Revealing the organism homeostasis mechanism. Fish. Shellfish Immunol. 92, 438–449. 10.1016/j.fsi.2019.06.037 31229644

[B16] Ghisaura S. Pagnozzi D. Melis R. Biosa G. Slawski H. Uzzau S. (2019). Liver proteomics of gilthead sea bream (*Sparus aurata*) exposed to cold stress. J. Therm. Biol. 82, 234–241. 10.1016/j.jtherbio.2019.04.005 31128654

[B17] Gracey A. Y. Fraser E. J. Li W. Z. Fang Y. X. Taylor R. R. Rogers J. (2004). Coping with cold: An integrative, multitissue analysis of the transcriptome of a poikilothermic vertebrate. Proc. Natl. Acad. Sci. U. S. A. 101 (48), 16970–16975. 10.1073/pnas.0403627101 15550548 PMC534716

[B18] Handelsman J. (2004). Metagenomics: Application of genomics to uncultured microorganisms. Microbiol. Mol. Biol. Rev. 68 (4), 195–685. 10.1128/MMBR.69.1.195.2005 PMC539003 15590779

[B19] Hassenrück C. Reinwald H. Kunzmann A. Tiedemann I. Gärdes A. (2020). Effects of thermal stress on the gut microbiome of juvenile milkfish (*Chanos chanos*). Microorganisms 9 (1), 5. 10.3390/microorganisms9010005 PMC7822048 33375015

[B20] Hazel J. R. Prosser C. L. (1974). Molecular mechanisms of temperature compensation in poikilotherms. Physiol. Rev. 54 (3), 620–677. 10.1152/physrev.1974.54.3.620 4366928

[B21] Hocine S. Singer R. H. Grunwald D. (2010). RNA processing and export. Cold Spring Harb. Perspect. Biol. 2 (12), a000752. 10.1101/cshperspect.a000752 20961978 PMC2982171

[B22] Hsieh S. L. Chen Y. N. Kuo C. M. (2003a). Physiological responses, desaturase activity, and fatty acid composition in milkfish (*Chanos chanos*) under cold acclimation. Aquaculture 220 (1–4), 903–918. 10.1016/S0044-8486(02)00579-3

[B23] Hu J. R. Wang G. X. Sun Y. P. Zhao H. X. Huang Y. H. Cao J. M. (2019). Effects of dietary sodium selenite and selenoyeast on growth performance, antioxidant responses and low temperature stress resistance of juvenile yellow catfish (*Pelteobagrus fulvidraco*). J. Fish. China 43 (11), 2394–2404. 10.11964/jfc.20181011479

[B24] Hu J. W. You F. Wang Q. Weng S. D. Liu H. Wang L. J. (2014). Transcriptional responses of olive flounder (*Paralichthys olivaceus*) to low temperature. PLoS One 9 (10), e108582. 10.1371/journal.pone.0108582 25279944 PMC4184807

[B25] Huyben D. Sun L. Moccia R. Kiessling A. Dicksved J. Lundh T. (2018). Dietary live yeast and increased water temperature influence the gut microbiota of rainbow trout. J. Appl. Microbiol. 124 (6), 1377–1392. 10.1111/jam.13738 29464844

[B26] Ibarz A. Blasco J. Beltrán M. Gallardo M. A. Sánche J. Sala R. (2005). Cold-induced alterations on proximate composition and fatty acid profiles of several tissues in gilthead sea bream (*Sparus aurata*). Aquaculture 249 (1-4), 477–486. 10.1016/j.aquaculture.2005.02.056

[B27] Ibarz A. Fernández-Borràs J. Blasco J. Gallardo M. A. Sanchez J. (2003). Oxygen consumption and feeding rates of gilthead sea bream (sparus aurata) reveal lack of acclimation to cold. Fish. Physiol. Biochem. 29 (4), 313–321. 10.1007/s10695-004-3321-8

[B28] Jastroch M. Buckingham J. A. Helwig M. Klingenspor M. Brand M. D. (2007). Functional characterisation of UCP1 in the common carp: Uncoupling activity in liver mitochondria and cold-induced expression in the brain. J. Comp. Physiol. B 177, 743–752. 10.1007/s00360-007-0171-6 17576568

[B29] Jastroch M. Wuertz S. Kloas W. Klingenspor M. (2005). Uncoupling protein 1 in fish uncovers an ancient evolutionary history of mammalian nonshivering thermogenesis. Physiol. Genomics. 22 (2), 150–156. 10.1152/physiolgenomics.00070.2005 15886331

[B30] Jiao S. Nie M. M. Song H. B. Xu D. D. You F. (2020). Physiological responses to cold and starvation stresses in the liver of yellow drum (*Nibea albiflora*) revealed by LC-MS metabolomics. Sci. Total Environ. 715, 136940. 10.1016/j.scitotenv.2020.136940 32014771

[B31] Jin S. B. Hu Y. N. Fu H. T. Sun S. M. Jiang S. F. Xiong Y. W. (2020). Analysis of testis metabolome and transcriptome from the oriental river prawn (*Macrobrachium nipponense*) in response to different temperatures and illumination times. Comp. Biochem. Physiol. Part D. Genomics Proteomics. 34, 100662. 10.1016/j.cbd.2020.100662 32114312

[B32] Kampoli A. M. Tousoulis D. Briasoulis A. Latsios G. Papageorgiou N. Stefanadis C. (2011). Potential pathogenic inflammatory mechanisms of endothelial dysfunction induced by type 2 diabetesmellitus. Curr. Pharm. Des. 17 (37), 4147–4158. 10.2174/138161211798764825 22204375

[B33] Kang Y. J. (2020). “Proteomics and metabolomics analysis of rainbow trout (*Oncorhynchus mykiss*) liver responses to heat stress,” (Lanzhou: Gansu Agricultural University), 6. Ph.D.Thesis.

[B34] Karanova M. V. Andreev A. A. (2010). Free amino acids and reducing sugars in the freshwater shrimp gammarus lacustris (*Crustacea, Amphipoda*) at the initial stage of preparation to winter season. J. Evol. Biochem. Physiol. 46 (4), 335–340. 10.1134/S0022093010040010 20799603

[B35] Karanova M. V. (2006). Seasonal variations of free amino acids the content of body flows in the fresh-water mollusk Lymnaea stagnalis. Izv. Akad. Nauk. Ser. Biol. 6, 719–724. 17168469

[B36] Ke S. F. Tu Z. Y. Li Z. M. Zhao S. J. Liu D. F. Shi X. T. (2019). Effects of acute temperature changes on the swimming abilities and oxygen consumption of *Ptychobarbus kaznakovi* from the lancang river. J. Appl. Ichthyol. 35 (3), 755–761. 10.1111/jai.13861

[B37] Lane A. N. Fan T. W. M. (2015). Regulation of mammalian nucleotide metabolism and biosynthesis. Nucleic Acids Res. 43 (4), 2466–2485. 10.1093/nar/gkv047 25628363 PMC4344498

[B38] Lermen C. L. Lappe R. Crestani M. Vieira V. P. Gioda C. R. Schetinger M. R. C. (2004). Effect of different temperature regimes on metabolic and blood parameters of silver catfish *Rhamdia quelen* . Aquaculture 239 (1–4), 497–507. 10.1016/j.aquaculture.2004.06.021

[B39] Li L. C. Li Q. Long Y. Cui Z. B. (2012). Microarray analysis of temperature stress effects on transcriptional expression in zebrafish larvae. Acta Hydrobiol. Sin. 36 (5), 882–891. 10.3724/SP.J.1035.2012.00882

[B40] Li X. Y. Zheng S. X. Wu G. Y. (2020). Nutrition and metabolism of glutamate and glutamine in fish. Amino Acids 52 (5), 671–691. 10.1007/s00726-020-02851-2 32405703

[B41] Lu J. G. Zheng M. Zheng J. J. Liu J. Liu Y. Z. Peng L. N. (2015). Transcriptomic analyses reveal novel genes with sexually dimorphic expression in yellow catfish (*Pelteobagrus fulvidraco*) brain. Mar. Biotechnol. 17 (5), 613–623. 10.1007/s10126-015-9650-z PMC4540775 26242754

[B42] Lucas A. (1996). “Physical concepts of bioenergetics,” in Bioenergetics of aquatic animals, englished. Editor Lucas A. (France: Taylor & Francis).

[B43] Lv H. R. Liu Y. L. Li H. D. Yin X. L. Wang P. Qu X. Y. (2021). Modulation of antioxidant enzymes, heat shock protein, and intestinal microbiota of large yellow croaker (*Larimichthys crocea*) under acute cold stress. Front. Mar. Sci. 8, 725899. 10.3389/fmars.2021.725899

[B44] MacMillan H. A. Knee J. M. Dennis A. B. Udaka H. Marshall K. E. Merritt T. J. S. (2016). Cold acclimation wholly reorganizes the Drosophila melanogaster transcriptome and metabolome. Sci. Rep. 6, 28999. 10.1038/srep28999 27357258 PMC4928047

[B45] Madaro A. Folkedal O. Maiolo S. Alvanopoulou M. Olsen R. E. (2018). Effects of acclimation temperature on cortisol and oxygen consumption in atlantic salmon (*Salmo salar*) post-smolt exposed to acute stress. Aquaculture 497, 331–335. 10.1016/j.aquaculture.2018.07.056

[B46] Magoč T. Salzberg S. L. (2011). Flash: Fast length Adjustment of short reads to improve genome assemblies. Bioinformatics 27 (21), 2957–2963. 10.1093/bioinformatics/btr507 21903629 PMC3198573

[B47] Melis R. Sanna R. Braca A. Bonaglini E. Cappuccinelli R. Slawski H. (2017). Molecular details on gilthead sea bream (*Sparus aurata*) sensitivity to low water temperatures from 1HNMR metabolomics. Comp. Biochem. Physiol. A Mol. Integr. Physiol. 204, 129–136. 10.1016/j.cbpa.2016.11.010 27872009

[B48] Meng X. H. Dong L. J. Shi X. L. Li X. P. Sui J. Luo K. (2019). Screening of the candidate genes related to low-temperature tolerance of *Fenneropenaeus chinensis* based on high-throughput transcriptome sequencing. PLoS One 14 (4), e0211182. 10.1371/journal.pone.0211182 30958828 PMC6453463

[B49] Miklos P. Katzman S. M. Cech J. J. (2003). Effect of temperature on oxygen consumption of the leopard shark, Triakis Semifasciata. Environ. Biol. Fishes 66 (1), 15–18. 10.1023/A:1023287123495

[B50] Morash A. Kajimura M. McClelland G. (2008). Intertissue regulation of carnitine palmitoyltransferase I (CPTI): Mitochondrial membrane properties and gene expression in rainbow trout (*Oncorhynchus mykiss*). Biochim. Biophys. Acta 1778 (6), 1382–1389. 10.1016/j.bbamem.2008.02.013 18359285

[B51] Neuman C. Hatje E. Zarkasi K. Z. Smullen R. Bowman J. P. Katouli M. (2016). The effect of diet and environmental temperature on the faecal microbiota of farmed tasmanian atlantic salmon (*Salmo salar* L.). Aquac. Res. 47 (2), 660–672. 10.1111/are.12522

[B52] Patti G. J. Yanes O. Siuzdak G. (2012). Innovation: Metabolomics: The apogee of the omics trilogy. Nat. Rev. Mol. Cell Biol. 13 (4), 263–269. 10.1038/nrm3314 22436749 PMC3682684

[B53] Phillips D. Covian R. Aponte A. M. Glancy B. Taylor J. F. Chess D. (2012). Regulation of oxidative phosphorylation complex activity: Effects of tissue-specific metabolic stress within an allometric series and acute changes in workload. Am. J. Physiol. Regul. Integr. Comp. Physiol. 302, R1034–R1048. 10.1152/ajpregu.00596.2011 22378775 PMC3362144

[B54] Qian B. Y. Xue L. Y. (2016). Liver transcriptome sequencing and de novo nnnotation of the large yellow croaker (*Larimichthy crocea*) under heat and cold stress. Mar. Genomics 25, 95–102. 10.1016/j.margen.2015.12.001 26683592

[B55] Qiang J. Zhong C. Y. Bao J. W. Liang M. Liang C. Li H. X. (2019). The effects of temperature and dissolved oxygen on the growth, survival and oxidative capacity of newly hatched hybrid yellow catfish larvae *(tachysurus fulvidraco♀ × pseudobagrus vachellii♂)* . J. Therm. Biol. 86, 102436. 10.1016/j.jtherbio.2019.102436 31789232

[B56] Ren X. Y. Yu Z. X. Xu Y. Zhang Y. B. Mu C. M. Liu P. (2020). Integrated transcriptomic and metabolomic responses in the hepatopancreas of kuruma shrimp (*Marsupenaeus japonicus*) under cold stress. Ecotoxicol. Environ. Saf. 206, 111360. 10.1016/j.ecoenv.2020.111360 32979723

[B57] Röhrig F. Schulze A. (2016). The multifaceted roles of fatty acid synthesis in cancer. Nat. Rev. Cancer 16 (11), 732–749. 10.1038/nrc.2016.89 27658529

[B58] Rong X. (2012). “Impacts of environmental factors on the intestinal micromicrobiota of apositchopus japonicus and its association with bacterial diseases,” (Qingdao: Ocean University of China). Ph. D thesis.

[B59] Sacristán H. J. Ansaldo M. Franco-Tadic L. M. Gimenez A. V. F. Greco L. S. L. (2016). Long-term starvation and posterior feeding effects on biochemical and physiological responses of midgut gland of *Cherax quadricarinatus* juveniles (*Parastacidae*). PLoS One 11 (3), e0150854. 10.1371/journal.pone.0150854 27018793 PMC4809490

[B60] Saito K. Matsuda F. (2010). Metabolomics for functional genomics, systems biology, and biotechnology. Annu. Rev. Plant Biol. 61, 463–489. 10.1146/annurev-arplant-043008.092035 19152489

[B61] Sanahuja I. Fernández-Alacid L. Sánchez-Nuño S. Ordóñez-Grande B. Ibarz A. (2019). Chronic cold stress alters the skin mucus interactome in a temperate fish model. Front. Physiol. 9, 01916. 10.3389/fphys.2018.01916 PMC6336924 30687126

[B62] Saraste M. (1999). Oxidative phosphorylation at the fin de siècle. Science 283 (5407), 1488–1493. 10.1126/science.283.5407.1488 10066163

[B63] Schryver P. D. Vadstein O. (2014). Ecological theory as a foundation to control pathogenic invasion in aquaculture. ISME J. 8 (12), 2360–2368. 10.1038/ismej.2014.84 24892581 PMC4260705

[B64] Silva J. E. (2003). The thermogenic effect of thyroid hormone and its clinical implications. Ann. Intern. Med. 139 (3), 205–213. 10.7326/0003-4819-139-3-200308050-00010 12899588

[B65] Sinaiko A. R. Caprio S. (2012). Insulin resistance. J. Pediatr. 161 (1), 11–15. 10.1016/j.jpeds.2012.01.012 22336575 PMC3357457

[B66] Soriano E. L. Ramírez D. T. Araujo D. R. Gómez-Gil B. Castro L. I. Sánchez C. G. (2018). Effect of temperature and dietary lipid proportion on gut microbiota in yellowtail kingfifish *Seriola lalandi* juveniles. Aquaculture 497, 269–277. 10.1016/j.aquaculture.2018.07.065

[B67] Stackebrandt E. Goebel B. M. (1994). Taxonomic note: A place for DNA-DNA reassociation and 16S rRNA sequence analysis in the present species definition in bacteriology. Int. J. Syst. Evol. Microbiol. 44 (4), 846–849. 10.1099/00207713-44-4-846

[B68] Sun L. T. Chen G. R. Chang C. F. (1995). Acute responses of blood parameters and comatose effects in salt-acclimated tilapias exposed to low temperatures. J. Therm. Biol. 20 (3), 299–306. 10.1016/0306-4565(94)00066-R

[B69] Sun Z. Z. Tan X. H. Xu M. L. Liu Q. Y. Ye H. Q. Zou C. Y. (2019). Liver transcriptome analysis and de novo annotation of the orange-spotted groupers (*Epinephelus coioides*) under cold stress. Comp. Biochem. Physiol. Part D. Genomics Proteomics 29, 264–273. 10.1016/j.cbd.2018.12.008 30641323

[B70] Tan K. Zhang B. Zhang H. K . Ma H. Y. Li Sh. K. Zheng H. P. (2020). Enzymes and non-enzymatic antioxidants responses to sequential cold stress in polymorphic noble scallop *Chlamys nobilis* with different total carotenoids content. Fish. Shellfish Immunol. 97, 617–623. 10.1016/j.fsi.2019.12.063 31870968

[B71] Voet D. Voet J. G. (1995). Biochemistry. 2nd edn. New York: Wiley.

[B72] Wang Q. Garrity G. M. Tiedje J. M. Cole J. R. (2007). Naive bayesian classifier for rapid assignment of rRNA sequences into the new bacterial taxonomy. Appl. Environ. Microbiol. 73 (16), 5261–5267. 10.1128/AEM.00062-07 17586664 PMC1950982

[B73] Wang Q. Tan X. Jiao S. You F. Zhang P. J. (2014). Analyzing cold tolerance mechanism in transgenic zebrafish (*Danio rerio*). PLoS One 9 (7), e102492. 10.1371/journal.pone.0102492 25058652 PMC4109919

[B74] Wang Z. Dong Z. D. Yang Y. T. Wang J. Yang T. H. Chen X. (2022). Histology, Physiology, and glucose and lipid metabolism of *Lateolabrax maculatus* under low temperature stress. J. Therm. Biol. 104, 103161. 10.1016/j.jtherbio.2021.103161 35180956

[B75] Wang Z. L. Qu Y. X. Yan M. Li J. Y. Zou J. X. Fan L. F. (2019). Physiological responses of pacifc white shrimp *Litopenaeus vannamei* to temperature fluctuation in low-salinity water. Front. Physiol. 10, 1025. 10.3389/fphys.2019.01025 31456695 PMC6700251

[B76] Weckwerth W. (2003). Metabolomics in systems biology. Annu. Rev. Plant Biol. 54, 669–689. 10.1146/annurev.arplant.54.031902.135014 14503007

[B77] Woo N. Y. S. (1990). Metabolic and osmoregulatory changes during temperature acclimation in the red sea bream, chrysophrys major : Implications for its culture in the subtropics. Aquaculture 87, 197–208. 10.1016/0044-8486(90)90275-R

[B78] Wu D. L. Huang Y. H. Chen Q. Jiang Q. Ch. Li Y. M. Zhao Y. L. (2019). Effects and transcriptional responses in the hepatopancreas of red claw crayfish *Cherax quadricarinatus* under cold stress. J. Therm. Biol. 85, 102404. 10.1016/j.jtherbio.2019.102404 31657746

[B79] Xu D. D. You Q. C. Chi C. F. Luo S. Y. Song H. B. Lou B. (2018). Transcriptional response to low temperature in the yellow drum (*Nibea albiflflora*) and identification of genes related to cold stress. Comp. Biochem. Physiol. Part D. Genomics Proteomics 28, 80–89. 10.1016/j.cbd.2018.07.003 30005389

[B80] Xu Y. Q. Zheng Y. M. Li W. F. Liu Y. Q. Ding Z. K. (2020). Gene expression, antioxidation and growth were considerably promoted by feeding dietary vitamin E and alanyl-glutamine dipeptide supplementation in juvenile Tilapia in cold freshwater. Aquac. Nutr. 26 (6), 2159–2168. 10.1111/anu.13154

[B81] Zhang D. Z. Liu J. Qi T. T. Ge B. M. Liu Q. N. Jiang S. H. (2018). Comparative transcriptome analysis of eriocheir japonica sinensis response to environmental salinity. PLoS One 13, e0203280. 10.1371/journal.pone.0203280 30192896 PMC6128516

[B82] Zheng Z. M. Jin C. H. Li M. Y. Bai P. F. Dong S. L. (2008). Effects of temperature and salinity on oxygen consumption and ammonia excretion of juvenile miiuy croaker, *Miichthys miiuy* (basilewsky). Aquacult. Int. 16 (6), 581–589. 10.1007/s10499-008-9169-7

